# Disease-specific classification using deconvoluted whole blood gene expression

**DOI:** 10.1038/srep32976

**Published:** 2016-09-06

**Authors:** Li Wang, William K. Oh, Jun Zhu

**Affiliations:** 1 Icahn Institute for Genomics and Multiscale Biology, Icahn School of Medicine at Mount Sinai, NY, 10029, USA; 2 Department of Genetics and Genomic Sciences, Icahn School of Medicine at Mount Sinai, NY, 10029, USA; 3 The Tisch Cancer Institute, Division of Hematology and Medical Oncology, Icahn School of Medicine at Mount Sinai, NY, 10029, USA

## Abstract

Blood-based biomarker assays have an advantage in being minimally invasive. Diagnostic and prognostic models built on peripheral blood gene expression have been reported for various types of disease. However, most of these studies focused on only one disease type, and failed to address whether the identified gene expression signature is disease-specific or more widely applicable across diseases. We conducted a meta-analysis of 46 whole blood gene expression datasets covering a wide range of diseases and physiological conditions. Our analysis uncovered a striking overlap of signature genes shared by multiple diseases, driven by an underlying common pattern of cell component change, specifically an increase in myeloid cells and decrease in lymphocytes. These observations reveal the necessity of building disease-specific classifiers that can distinguish different disease types as well as normal controls, and highlight the importance of cell component change in deriving blood gene expression based models. We developed a new strategy to develop blood-based disease-specific models by leveraging both cell component changes and cell molecular state changes, and demonstrate its superiority using independent datasets.

Blood-based biomarker assays are minimally invasive and can be easily implemented in clinical practice. As such, diagnostic and prognostic models built on peripheral blood gene expression have been reported for various types of diseases^1^,^2^,^3^,^4^,^5^. However, several important issues remain to be addressed. First, most of the previous studies focused on only one disease type. It is hard to know whether the identified gene expression signature represents a disease-specific effect or a more common physiological change. This concern is particularly relevant for blood biomarker-based disease modeling since the peripheral blood communicates with most other tissues in the human body^6^ and can be affected by a wide-range of pathological and/or physiological conditions^7^. Second, whole blood gene expression represents a mixture of hematopoietic cells, and is greatly influenced by the cell type frequency. Multiple computational methods have been developed to deconvolute whole blood gene expression into cell frequency and cell type-specific gene expression^8^,^9^,^10^,^11^,^12^. When applied to a specific disease, blood gene expression signatures attributable to a physiological change can be decomposed into cell frequency changes and cell molecular state changes. However, most of these existing gene expression deconvolution methods, such as csSAM^13^ and PSEA^14^, are designed mainly for hypothesis testing or estimating the group difference between cases and controls. Thus, it remains to be explored how to develop robust sample-wise disease classification based on cell-specific gene expression change, and more importantly, whether prediction accuracy can be improved by considering cell-type specific gene expression changes.

To address these issues, we carried out a meta-analysis of 46 whole blood gene expression datasets covering a wide range of diseases or physiological conditions. Our analysis shows a striking overlap of signature genes shared by multiple diseases, which is driven by the underlying common patterns of cell component change. These observations suggest the necessity to develop disease-specific classifiers that can distinguish different disease types as well as normal controls. To build such models, we developed a new classification strategy that can take into consideration of both cell component changes and cell molecular stage changes. Specifically, we deconvoluted the original gene expression profile into a cell component profile and a residual expression profile for each sample, and built classifiers based on these deconvoluted features. By testing independent datasets, we show that the classifiers with incorporated cell component profiles and residual expression profiles performed significantly better than those without. Both the assembled datasets and the algorithms developed can be found in the R package *declassification* (http://research.mssm.edu/integrative-network-biology/Software.html).

## Results

### Remarkable similarity observed among multiple diseases in whole blood gene expression profiles

**Figure 1:**
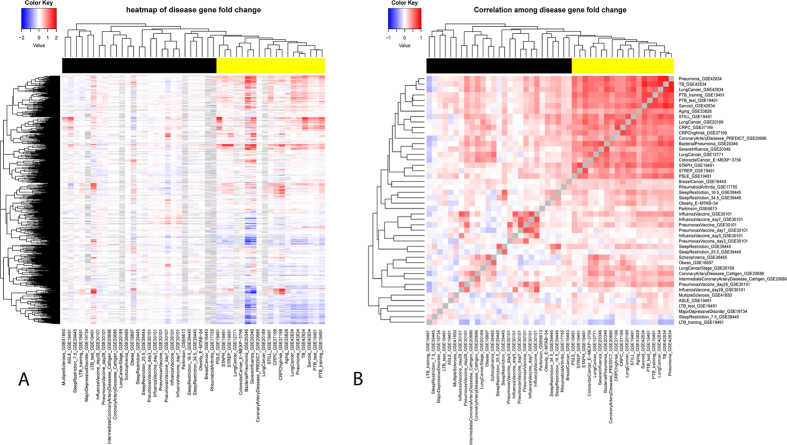
(**A**) Fold change profiles of 3161 disease informative disease genes across the 46 datasets. Rows represent genes, and columns represent datasets. Each cell represents the log2 fold change of the corresponding gene in the corresponding dataset, which was calculated by comparing the gene expression in case samples with that in control samples. For display purpose, cells with value >2 (<−2) were set to 2 (−2). Cells of the grey color indicate the data were not available. (**B**) Correlation matrix of disease datasets. Each cell represents the Spearman’s correlation coefficient of the gene fold change profile (as shown in A) between two datasets. In both (**A**) and (**B**), disease datasets were clustered using complete-linkage hierarchical clustering. The distance matrix used in hierarchical clustering was calculated as 1 - the correlation matrix as shown in (**B**).

A total of 46 whole blood gene expression datasets were collected from public databases (Table 1). These covered a wide range of disease types including infectious diseases, metabolic diseases, neurodegenerative diseases and multiple cancers. Some less morbid physiological conditions were also included, e.g., aging, sleep restriction and vaccinations (for simplicity, these are referred to as ‘disease datasets’ hereafter). For each dataset, we obtained the gene expression fold change profile by comparing the case samples with the control samples in that dataset. Figure 1A shows the fold change profiles of 3161 disease informative genes across the 46 datasets (see Methods for the definition of disease informative genes). Similarity matrix among the 46 datasets (Fig. 1B) was then derived based on the correlation of their profiles in Fig. 1A. The above analysis revealed a tight cluster consisting of 19 datasets which show remarkable similarity between each other (yellow color in the color bar of Fig. 1).

The tight cluster in Fig. 1 includes datasets of different disease types as well as those generated on different array platforms. We then assessed whether potential confounding factors drove this cluster. We adjusted gene expression data for the age, gender and race of the patients when available (Table S1 lists whether detailed age, gender and race information is available for each dataset), and re-derived the expression change profiles. When the adjusted profiles were used, the correlation among the 19 datasets remained notably higher than that of the others although the clustering structure slightly changed (Figure S1). The observation is consistent with the fact that most of these datasets used age, gender and race matched controls. We also obtained median age, gender proportion and race proportion for case samples in each dataset (Table S2), and then compared the summary demographic characteristic for datasets inside and out of the tight cluster. None of the above characteristics showed significant differences between datasets inside and out of the cluster (Wilcoxon rank sum test p = 0.68, 0.19 and 1 for median age, gender proportion and race proportion, respectively). Similarly, there was no significant correlation between the array platform used and whether the dataset was inside the cluster (Fisher’s exact test p = 0.13). In addition, if only the 25 datasets generated on “Illumina HumanHT-12” are used, datasets in the tight cluster in Fig. 1 were clustered together similarly (Figure S2). Interestingly, there was a significant association between the cluster and the disease category (Table S3, Fisher’s exact test p = 0.0031). Specifically, inflammatory and infectious disease datasets were overrepresented in the cluster with marginal significance (Odds Ratio = 4.1, Fisher’s exact test p = 0.08). Cancer datasets were also overrepresented, although this was not statistically significant (Odds Ratio = 5.14, Fisher’s exact test p = 0.18). On the other end, neuronal disease datasets were underrepresented (Odds Ratio = 0, Fisher’s exact test p = 0.033). Non-morbid conditions, i.e., vaccine and sleep deprivation, were also underrepresented (Odds Ratio = 0, p = 0.083). In summary, we observed a common blood gene expression pattern shared by a spectrum of disease types, especially inflammatory conditions, infectious diseases and cancer. This common pattern was not caused by the effect of patient characteristics such as age, gender, race or the array platform.

It is notable that the dataset similarity in Fig. 1 was measured based on 3161 disease informative genes pooled from the 46 datasets. These genes were selected to reduce non-informative genes while encouraging equal contributions from each dataset (see Methods for details). The fold changes of these genes across datasets can be found in Table S4. As expected, these genes were enriched in interferon signaling, cytokine signaling and other immune pathways (Table S5). The clustering structure did not depend on the selection of the 3161 disease informative genes. When dataset similarity was measured by 7,522 disease informative genes (assembled from top 300 differentially expressed genes in each dataset) or 10398 disease informative genes (top 500 genes), the clustering structure was similar, and most if not all of the previous 19 datasets remained in the tight cluster (Figure S3).

Indeed, these 19 datasets overlap significantly in their differentially expressed genes (FDR < 0.1, Figures S4 and S5). To further investigate these common signature genes, we identified 417 and 630 commonly up and down-regulated genes (CUG and CDG), respectively, that were shared by more than 10 out of the 19 datasets (corresponding to FDR = 0.002 and 0.004, respectively; see Methods for details). These commonly regulated genes (Table S6) could potentially be utilized in future blood biomarker studies to distinguish disease-specific signatures from more common effects. For example, we analyzed a dataset published this year after the above analysis was done: a longitudinal whole blood transcriptome study of systemic lupus erythematosus (SLE)^15^. The dataset consisted of 924 samples from 158 pediatric SLE patients followed longitudinally for up to 4 years, as well as 72 samples of healthy pediatric individuals as controls (GSE65391). The patient samples were categorized into three groups based on disease activity (DA). The heatmap of the CUGs and the CDGs indicates that the expression of these genes consistently changed in the SLE dataset (Figure S6): up-regulation of CUGs and down-regulation of CDGs in SLE patients as compared to controls. In fact, there was a striking overlap between the CUG/CDG and the differentially expressed transcripts (DETs) between SLE patients and healthy controls as identified by authors of the study. 22% of the up-regulated DETs overlap with CUGs (Odds Ratio = 39.5, Fisher’s exact test p = 1e-243), and 18% down-regulated DETs overlap with CDGs (Odds Ratio = 48.1, Fisher’s exact test p < 1e-256). Among patients with different disease activities, there was a trend of up-regulation of CUG and down-regulation of CDG as disease activity accelerated: 26% of up-regulated DETs in DA3 group as compared to DA1 group overlapped with CUG (Odds Ratio = 20, Fisher’s exact test p = 5e-58), and 15% down-regulated DETs in DA3 group overlapped with CDG (Odds Ratio = 5.7, Fisher’s exact test p = 3e-5). Therefore, a significant portion of the SLE disease signature genes and disease activity signature genes identified by the new study^15^ were not disease specific but commonly regulated in a range of different diseases. As expected, this new dataset was clustered into the tight cluster in Fig. 1 together with the other 19 datasets (Figure S7).

### The underlying common patterns of cell component change

**Figure 2:**
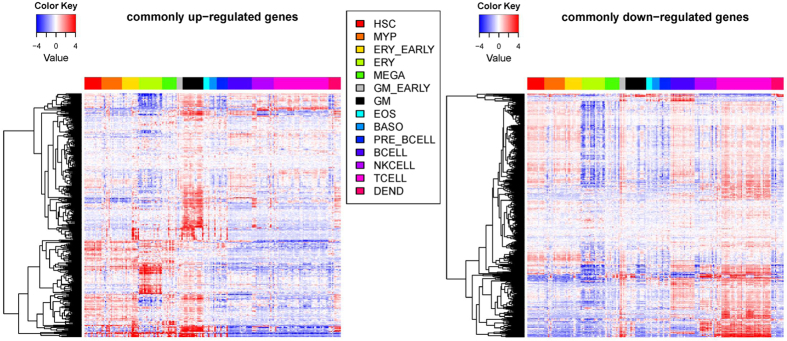
Heatmap of commonly regulated genes across different types of blood cell lines. Rows represent up-regulated genes (left) or down-regulated genes (right). Columns represent blood cell lines which are grouped according to the lineage (column legend). Some abbreviations: HSC: Hematopoietic stem cell. MYP: myeloid progenitor. ERY: Erythroid cell. MEGA: megakaryocyte. GM: Granulocyte/monocyte. EOS: eosinophil,BASO: basophil. DEND: dendritic cell.

Given a mixture of cells in blood, we first studied the cellular sources of these common signature genes. We plotted their gene expression across a panel of hematopoietic cells of different lineages^16^ (Fig. 2). Intriguing patterns of cell type-specific overexpression were observed: the up-regulated genes were consistently more likely to be over-expressed in myeloid cells, e.g., granulocytes/monocytes (GM) and erythroid cells (ERY), while the down-regulated genes were more like to be overexpressed in lymphocytes, e.g., B cells and T cells. This observation suggests there might be common cell frequency changes among these various diseases, i.e., up-regulation of myeloid cells and down-regulation of lymphoid cells.

Since cell count data was not available for most of these datasets, we used computational deconvolution methods to infer their cell frequency or cell component profile. Existing methods to infer cell components can be grouped into two categories based on whether cell markers or cell signature matrix data are needed as inputs^17^. To reduce the algorithm-specific bias on the results, we chose one algorithm from each category, i.e., DSA^8^ and CIBERSORT^9^. In addition, the input data used by the two algorithms are very different; thus we tested whether the results are dependent on a particular data input or not. The cell marker data for the DSA algorithm included 8 types of hematopoietic cells^18^, while the CIBERSORT signature matrix represented 22 types/subtypes ofleukocytes^19^. The former lacked detailed categorization of cells at different status, but included erythroblasts and megakaryocytes which were missing from the latter. Despite all these differences, the estimated cell frequency from the two methods correlated reasonably well with each other (Figure S8, the mean correlation coefficient is 0.61)

**Figure 3:**
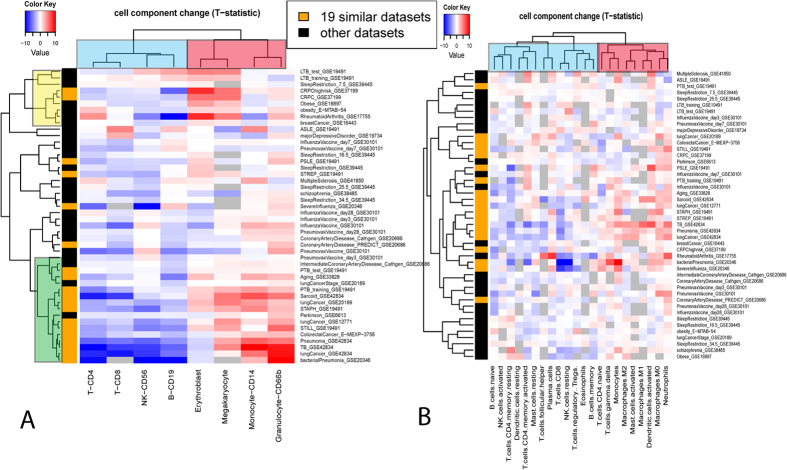
Heatmap of cell component change profiles. The cell frequency was estimated by DSA (**A**) and CIBERSORT (**B**). Each row represents a dataset, and the row side color indicates members of the 19 similar datasets (orange) and the others (black). Each column represents a different cell component. The color in each cell of the heatmap encodes T-statistics in testing the cell component difference between the case and the control groups in each dataset. Complete-linkage clustering was applied with distance = 1-pearson’s correlation of two profiles. Grey color indicates that the T-statistics is not calculable. In this analysis, it corresponds to the situation where the estimated proportion of that particular cell component is zero for all samples in the dataset.

We then calculated how different cell components changed in each disease dataset. As shown in Fig. 3, the cell components were clearly clustered into two groups. One group (red cluster) composed mainly of cells from myeloid lineages was predominantly up-regulated among the different diseases. The other group (blue cluster) mainly composed of cells from lymphocyte lineages was predominantly down-regulated. Such a pattern was revealed by the results of both DSA and CIBERSORT. When the 19 similar datasets identified above were analyzed, the pattern was even more striking. For example, according to the DSA results, Granulocytes, Megakaryocytes, Monocytes and Erythroblasts were significantly up-regulated (P-value < 0.05) in 74%, 53%, 53% and 37% of the 19 datasets, while CD4 T-cell, B-cell, CD8 T-cell and NK were significantly down-regulated in 84%, 74%, 53% and 53%. Thus, deconvolution analyses confirmed the common patterns of cell frequency change among disparate diseases.

Figure 3 also reveals deconvolution algorithm-specific patterns, which demonstrated intriguing results. There are two clusters in Fig. 3A, the yellow cluster with Erythroblasts and Megakaryocytes that are up-regulated compared to Monocytes and Granulocytes, and the green cluster with Monocytes and Granulocytes that are up-regulated. In addition, datasets of the same disease type tended to belong to the same of two clusters. For instance, the three datasets of lung cancer were all within the green cluster. The two datasets of obesity were within the yellow cluster. This indicates that the clustering is biologically relevant rather than an artefactual effect. Thus, the cell component change profile may help reveal a phenotypic linkage between different diseases. For instance, there are four types of cancers among the datasets. While castration-resistant prostate and breast cancers are grouped together with obesity in the yellow cluster, lung and colon cancers are grouped together with pneumonia in the green cluster. In the literature, there is a well-documented linkage between obesity and either breast cancer or prostate cancer, but much less can be found between obesity and lung or colon cancer. However, such clustering cannot be seen in the CIBERSORT results, most likely because the input signature matrix for CIBERSORT doesn’t contain erythroblasts and megakaryocytes.

A recent study using CIBERSORT to infer leukocyte representation in bulk tumor transcriptomes identified associations between leukocyte subsets and cancer survival^19^. There is remarkable resemblance between those results and our current report. Cells of lymphocyte lineages are generally associated with favorable survival outcome, while cells of myeloid lineages are associated with adverse survival outcome. The resemblance can even been seen for cells within a particular state. For instance, activated dendritic cells behave similarly to other myeloid cells, while resting dendritic cells behave more like lymphocytes. Therefore, the common pattern of cell component change is not limited to peripheral blood, but can be extended to tumor-infiltrating immune cells of multiple cancers. On the other hand, obvious differences were also observed. While T-cell gamma delta ranks the highest in terms of its association with favorable tumor survival, it was clustered closer to myeloid cells in our dataset. Whether this indicates different functionality of T-cell gamma delta in peripheral blood and tumors requires further study.

**Figure 4:**
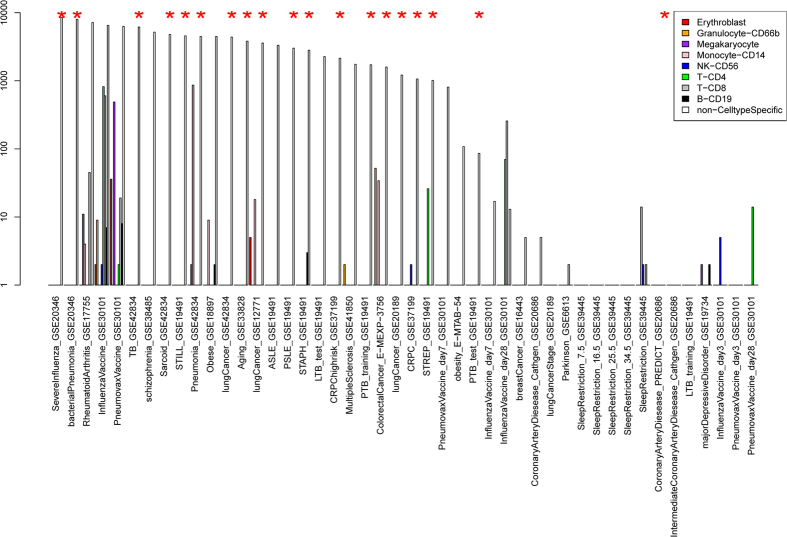
Numbers of differentially expressed genes output from csSAM algorithm and SAM algorithm (FDR < 0.1). Datasets annotated with the red star represent the 19 similar disease datasets in Fig. 1.

To further demonstrate that cell component change is the true underlying cause for the observed disease similarity, we carried cell type-specific differential expression analysis using the csSAM algorithm^13^. Figure 4 shows the number of differentially expressed genes using csSAM and traditional SAM algorithms^20^ (FDR < 0.1). While there are thousands of genes differentially expressed using traditional SAM analysis for the aforementioned 19 common datasets, the number of differentially expressed genes in a given cell type is much smaller (note that the y-axis is in log scale). More importantly, the tight cluster observed in Fig. 1 disappears when the dataset similarity was calculated based on cell type-specific expression change profiles (Figure S9). All of this evidence thus supports the notion that common patterns of cell component change drive the similarity of disease signature genes.

### Using deconvolution-based classification strategy to build disease-specific classifiers

Given the prevailing common signature genes across multiple diseases, we aimed to build disease-specific classifiers which can distinguish different disease types as well as normal controls. When training the classifier for a particular disease type, we made use of the various disease datasets collected above, and included in the negative controls both samples from other disease types and normal controls.

**Figure 5:**
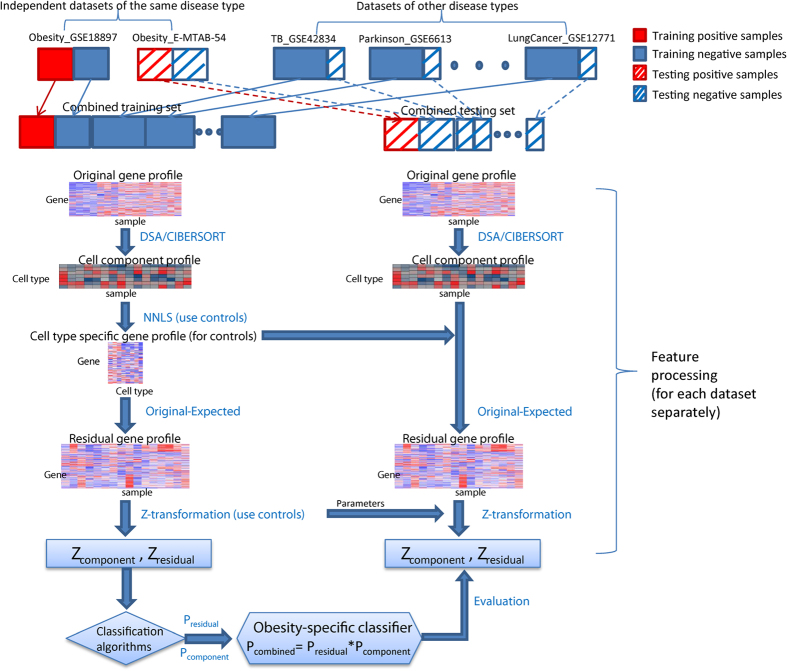
Workflow of training and validating disease-specific classifiers using deconvolution-based strategy.

To incorporate cell component deconvolution into a disease-specific classification, we developed a new strategy as illustrated in Fig. 5. This classification strategy consists of three main steps: 1) deconvoluting the original gene expression profile into the cell component profile and residual expression profile, 2) building classifiers based on the cell component profile and residual expression profile, separately, and 3) combining prediction results from the above two classifiers (see Methods for details). The core step involves calculating the residual expression profile for each sample. The residual gene expression profile was defined as the deviation of the observed gene expression value from the expected value given its current cell abundance and assuming it is under normal conditions. For a normal sample, the deviation is assumed to be caused by random noise. For a disease sample, it is assumed to be caused by cell type-specific differential expression under the disease condition (if there is any), plus the random noise. The expression deviation caused by pure cell frequency change won’t be reflected in the residual expression profile. Like csSAM or PSEA, the residual profile can distinguish cell-type specific differential expression from cell frequency change. However, unlike these existing methods which capture group-wise differences, the residual profile is defined per sample wise, which is essential for such a classification problem.

There are several technical details in our procedure that are worth mentioning. First, a significant concern of combining different gene expression datasets is comparability in terms of platform differences and variations in sample processing. To make datasets more comparable to each other, we performed gene-wise Z-transformation based on control samples in each dataset (see Methods for details). Second, we carefully designed our training and evaluation schema to make sure disease samples in the training and test sets originated from different datasets. Although Z-transformation can minimize dataset-specific effects, it was still possible for the disease-specific classifiers to capture dataset-specific artifact rather than real biological differences. For such considerations, we only constructed and evaluated classifiers for disease types of which there were more than one independent datasets. There were four such disease types, i.e., pneumonia, tuberculosis, lung cancer and obesity, corresponding to 10 datasets (datasets with asterisk* in Table 1).

**Figure 6:**
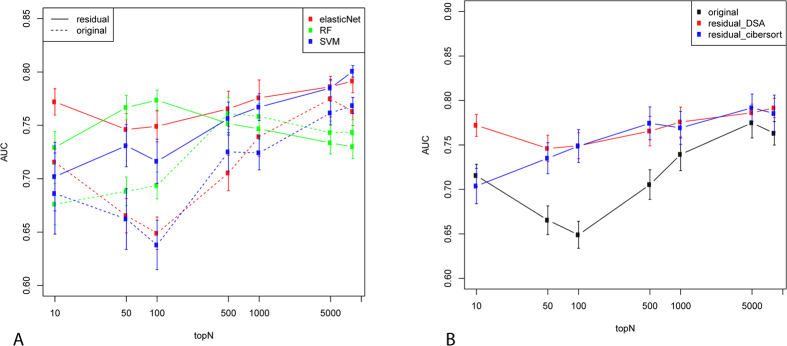
Performance of disease-specific classifiers based on the residual gene expression profiles or the original gene expression profiles when different classification algorithms were used (**A**) or when different cell deconvolution methods were used to estimate residual profiles. The Y-axis represents the average AUC score as assessed by the 10 independent datasets. The error bar was calculated by running the training and evaluation 10 times (see Methods for details of the sample splitting schema). The X-axis represent the number of top-ranking (based on t-statistics) genes preselected to be included in the classifiers.

We first built disease-specific classifiers based on the residual expression profiles only and compared them with the original expression profiles. Figure 6 plots the prediction accuracy as measured by the average area under the ROC curve (AUC) score of the 10 independent datasets. Genes were pre-filtered to remove low-expressed genes (see Methods). A simple feature selection strategy was used to include the top N genes in the classifiers (based on t-statistics in the training dataset). In most cases, the residual prediction profiles performed significantly better than the original expression profiles. This superiority did not depend on the particular classification algorithms used; Fig. 6A shows prediction performance using three different classification algorithms, i.e. elasticNet, SVM and Random Forest (RF). It also did not depend on the deconvolution methods used in inferring cell component profiles; Fig. 6B shows performance when the cell components were estimated by DSA and CIBERSORT, respectively. Finally, it is also notable that the advantage of residual profiles was more striking when the number of genes included in the classifiers (x-axis in Fig. 6) was smaller. Therefore, compared to the original expression profile, the residual profile performed considerably better by itself.

**Figure 7:**
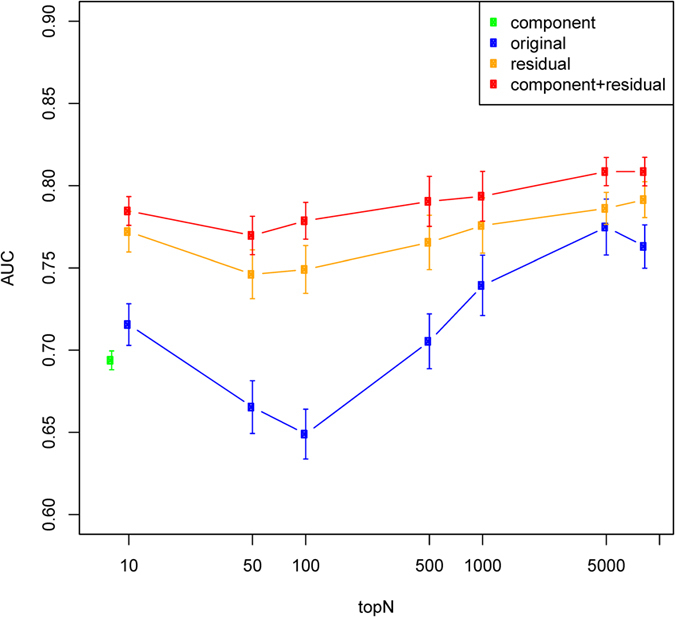
Performance of disease-specific classifiers when residual gene expression-based classifiers were combined with cell component-based classifiers. The figure legend is the same as Fig. 6.

We then combined the residual profile with the cell component profile in classification. As shown in Fig. 3A, the cell component profile appears to cluster datasets of the same disease type together, thus suggesting that it carries useful disease-specific signals. Since such information is independent of the residual expression profile, combining residual and cell component profiles could in theory boost classification accuracy. Indeed, we found this to be the case. We used a simple strategy in combining the two types of features. For each disease, we built two classifiers using each type of feature separately. To make a prediction, we multiplied the prediction result (posterior probability from elasticNet) of the two classifiers as the final prediction. As shown in Fig. 7, the combined classifier performed significantly better than each classifier alone. Therefore, our results suggest that the advantages of a deconvolution-based classification strategy come from two factors: the superiority of the residual profile itself compared to the original profile, and the effective combination of residual and cell component profile which appear to be complementary to each other.

Functional analysis of genes selected by the disease-specific classifiers suggests their relevance to the specific disease. Table S7 lists the genes selected by the above disease-specific classifiers (see Methods for details). Pathways enriched in these gene lists can be found in Table S8. Much evidence in the literature suggests an association between these pathways and their corresponding diseases. For instance, obesity-specific classifiers are enriched in IGF1R pathways^21^, and TB-specific classifiers are enriched for interferon gamma signaling pathways^22^. Genes included in pneumonia-specific classifiers are enriched in the glucose metabolism pathway. Previous studies have demonstrated that serum glucose levels can be used to predict death in patients for community acquired pneumonia^23^. Genes included in our lung cancer-specific classifiers are enriched for genes in redox sensor CtBP complex and CtBP complex has been shown to mediate hypoxia-induce tumor cell migration^24^.

## Discussion

To our knowledge, this is the first large-scale meta-analysis of whole blood gene expression across multiple diseases. Our analysis uncovered a significant overlap of signature genes among multiple diseases, which was driven by interesting common patterns of blood cell component change, specifically up-regulation of myeloid cells and down-regulation of lymphocytes. Similar patterns were seen in tumor infiltrating immune cells when comparing tumors with adverse outcomes to those with favorable outcomes. Because of the existence of such common immune response signatures, future studies of whole blood expression biomarkers should compare their particular signatures with other disease signatures to distinguish disease-specific effects from more common effects. Our assembled datasets will provide valuable resources for such comparative analyses.

We also developed a new deconvolution-based classification strategy in this study, and applied it to build disease-specific classifiers from blood gene expression. We demonstrated that gene deconvolution techniques can be effectively incorporated into the classification problem to improve prediction performance. The improvements are likely to result from multiple factors. First, similar to methods like csSAM, the residual expression profile is designed to remove the sample variance caused by cell frequency change, and thus is more powerful in detecting true physiological changes. Second, the cell component profile may be more robust than the original gene profile in capturing signals from cell frequency changes. If we analogize cell markers expressed in the same cell components to genes expressed in the same pathway, the cell component profiles inferred by deconvolution methods are like pathway-based features. It has been shown that such pathway-based features perform more robustly than single gene-based features^25^. Third, residual expression profiles are better in distinguishing different diseases compared to the original profile. The common gene signatures are shown to be mainly driven by the cell component changes. Having the effect of cell frequency change removed, the residual expression profiles are thus less likely to represent a common effect and more likely to represent a disease-specific phenomenon, which makes them better candidates to distinguish different disease states.

Although the deconvolution-based classification strategy was designed for whole-blood disease classification, some of its merits as illustrated above are not contingent to this particular platform. Thus, we believe it could be applicable to a wide range of classification problems where mixed samples are involved. For instance, tumors are well-known to be heterogeneous samples of various cell types. Our strategy is likely to be beneficial in building improved cancer diagnostic and prognostic models from tumor gene expression data.

We present here a successful strategy to incorporate deconvoluted gene expression into the disease classification problem. With advances in single cell sequencing technologies, it is possible to sort whole blood into different types of cells and then profile each cell individually. Compared with profiling individual cells by single cell sequencing, our approach is much easier to implement in common clinical practice. However, the residual expression profile resulting from deconvolution methods cannot tell the exact cell type from which the differential expression was derived. Newer methods will need to be developed to capture such information and improve prediction performance. Moreover, there are many more deconvolution techniques. Although we compared and applied several of them in this study, e.g., DSA versus CIBERSORT, non-negative least square versus ordinary least square, this comparison is far from exhaustive. Thus, the best performing deconvolution techniques for our classification strategy remain to be explored further.

A recent meta-disease analysis^26^ highlighted four circulating blood biomarkers—alpha-1-acid glycoprotein, albumin, VLDL particle size, and citrate—predictive of the short-term risk of death from all causes. According to their study, all four biomarkers were predictive of death from cancer and nonvascular causes in addition to cardiovascular mortality. Their analysis is based on molecular profiling of plasma samples by NMR spectroscopy which is a significantly different approach from mRNA profiling of whole blood samples by microarray as used in our study. However, it is interesting that both studies observed the presence of systemic biomarkers across diverse diseases. In addition, one of the four markers identified in their study, i.e., alpha-1-acid glycoprotein, is related to the commonly regulated genes identified in our study. The gene *ORM1*, whose protein product is alpha-1-acid glycoprotein, is specifically expressed in liver tissue, and is also expressed in monocytes. Although *ORM1* was not included into our commonly regulated gene list (since a very stringent cutoff of FDR < 0.01 is used), *ORM1* was up-regulated in 8 out of the 19 datasets in the tight cluster of Fig. 1 (FDR = 0.046). Future study is needed to further investigate the relationship of the two sets of potentially complementary systematic biomarkers.

In summary, we provide valuable data resources and insights for future studies of the whole blood gene transcriptome, as well as an effective deconvolution-based classification strategy that can have wider applications.

## Materials and Methods

### Whole blood gene expression profiling datasets

We searched public gene expression databases, i.e., GEO and ArrayExpress, for datasets consisting of a large number of whole blood gene expression profiles. We obtained a total of 18 datasets (>100 samples profiled each). From each dataset, we split samples in the dataset into case and control groups according to the experimental design and curating the phenotypic data provided. For instance, if multiple disease types were profiled in one dataset, we split the dataset into multiple datasets with one for each disease type. If a dataset was based on a longitudinal study, we split it into multiple datasets with one for each time point. In summary, we generated a total of 46 datasets as shown in Table 1. The processed gene expression data was downloaded for each dataset, and was further quantile normalized. The probe ID was mapped to gene symbol, and the expression value was averaged when multiple probes mapped to one gene symbol. For each dataset, genes with missing values for more than half of samples were removed, the rest missing values were inferred from R package *impute*. The column gene# in Table 1 shows the distinct number of gene symbols in each dataset.

### Similarity among disease gene expression profiles

To calculate similarity of disease gene expression profiles, we obtained the fold change of each gene as compared to the control group for each dataset. We defined a collection of disease informative genes, which consists of genes that are 1) among the top 100 most differentially expressed genes in at least one of the datasets, and 2) measured in more than half of the datasets. There are a total of 3161 genes in the disease informative gene collection. We applied the first criterion to exclude genes that are unchanged in any disease, and to reduce bias toward datasets with large numbers of differentially expressed genes. The second criterion was used to remove genes with too many missing values across the datasets. We then calculated the Spearman’s correlation of the fold change profile of genes in the collection between each pair of datasets.

The common differentially expressed genes among datasets were defined as following: we first obtained differentially expressed genes for each dataset using R package *limma* with FDR cutoff of 0.1. We then defined common differentially expressed genes as those shared by at least *n* number of datasets. We chose the cutoff of n based on the estimated FDR. The FDR was estimated by permutation as following. Assuming there are o_i_ differential genes shared by > = *n*_*i*_ datasets, and there are *p*_*i*_ genes shared under random permutation, the FDR was then calculated by *p*_*i*_*/o*_*i*_. The permutation was performed for each dataset separately while keeping the number of differential genes in each dataset the same.

### Analysis of potential confounding factors

Before deriving differentially expressed genes in each dataset, the effect of age, gender and race when available was adjusted by including them as covariates in the linear model using R package *limma*. We also assessed whether datasets inside and outside the tight cluster in Fig. 1 differ in patient characteristics or array platform. After excluding some longitudinal datasets (those with blank disease name in Table 1 except for Aging_GSE33828) to avoid multiple counts of the same disease datasets, there are 18 datasets inside the tight cluster and 15 datasets outside. Summary demographic information, i.e., median age, gender proportion and race proportion were calculated using case samples in each dataset or curated directly from the manuscript when information for individual sample is not available. Array platforms were categorized into three types, i.e, Illumina HumanHT (19 dataset), Affymetrix HG-U133 (6 datasets) and the others (8 datasets).

### Cell type-specific expression differences

We used R package *csSAM*^13^ to obtain cell type-specific expression differences between case and control for each disease dataset. We used the expression value without log-transformation as suggested^27^. As a direct comparison, we also conducted traditional SAM^20^ analysis between case and controls. FDR cutoff of 0.1 was applied in both cases to obtain significantly differentially expressed genes.

### Estimation of cell frequency

Recently, computational methods were reported for predicting fractions of multiple cell types in gene expression profiles of admixtures. Cell proportion estimation methodologies can be categorized into two main groups based on whether it relies on reference signature expression profiles of different cell subsets or known cell subset-specific marker genes. One exemplar method from the former category is DSA^8^. DSA models the cell mixing process through a linear model, and estimates cell type frequencies by solving the linear equations using cell type specific marker genes. A recently developed algorithm CIBERSORT^9^ represents the latter category, which requires an input matrix of reference gene expression signatures of different cell types. CIBERSORT applies linear support vector regression, a machine learning approach highly robust with respect to noise, to deconvolve the mixture. We implemented the DSA algorithm in R and utilized cell markers from HaemAtlas project^18^. The marker list represented by array probes were downloaded from R package *CellMix*^10^, and mapped to gene symbols. The *CIBERSORT* R package and its associated leukocyte signature matrix were utilized with all default parameters. For both DSA and CIBERSORT, we used the gene expression value without log-transformation.

### Residual gene expression profiles

We model gene expression as a linear combination of its expression in different cell types as following


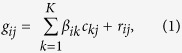


where 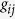 denotes observed mRNA expression of gene *i* in sample *j*, 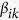 denotes the cell type-specific expression of gene *i* in cell type *k* under normal(non-disease) condition. 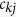 denotes the frequency of cell type *k* in sample *j*. If 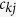 is not available from the experimental data, it can be computationally estimated as described above. And 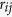 denotes residual expression of gene *i* in sample *j*. The residual expression represents the deviation of the observed expression from expected one given the current cell type frequency and given the sample is under normal condition. For control samples, 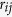 is caused by random noise. For disease samples, 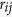 is the mixture of random noise and the biological difference between 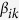 and 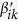, where 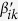 denotes the cell type-specific expression of gene *i* in cell type *k* under disease condition.

Since 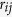 represents random noise within control samples, we can use control samples to estimate 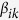. In particular, we used non-negative least square optimization implemented in *fcnnls* function of R package *NMF*. We used non-negative least square optimization (which performed much better than ordinary least square (Figure S10)):


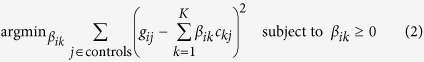


Given the estimated 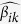 from formula (2), the estimated residual gene expression 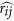 for both control and disease sample can be easily derived as following


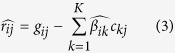


### Z-transformation

To makes different datasets more comparable while keeping the disease specific signals, we performed gene-wise Z-transformation for each feature before building any classifiers. Specifically, for each dataset, we scaled each gene separately according to its mean and standard deviation (SD) among the control samples, so that each genes in control samples will have an approximately standard normal distribution N(0, 1), while genes in the disease samples may deviate from standard normal distribution for disease related differential expression. The underlying assumption is that genes in the control samples should have similar distribution across different datasets, while genes in the disease samples could differ significantly from one dataset to another.

### Training and evaluating disease-specific classifiers

We selected 32 out of the 46 datasets in Table 1 to build disease specific classifiers. We excluded some datasets (those with the column of disease name blank in Table 1) mainly to ensure sample independence. For instance, we only select one time point with the most significant gene expression changes among the multiple time points for longitudinal studies. Similarly, we removed dataset CRPChighrisk_GSE37199 as the dataset is a subset of dataset CRPC_GSE37199. The aging dataset (Aging_GSE33828) was also removed because it is a less well-defined disease condition. As a result, there are a total of 2726 samples in the combined dataset including 1080 normal controls and 1646 disease samples covering 24 different disease types or subtypes.

### Splitting samples into training and testing sets

For evaluating the performance of the disease-specific classifiers, the datasets were split into training and testing datasets as illustrated in Fig. 5 and detailed as following. Traditional cross validation (CV) strategy biases to overestimation of the prediction performance since training and testing samples are drawn from the same datasets. To overcome the biase, we only selected disease types where there were more than one independent dataset, and trained and evaluated classifiers using different independent datasets. In each round of evaluation, one dataset (e.g., Obesity_GSE18897) was included in the combined testing set, and the other dataset(s) of the same disease type (e.g., Obesity_E-MTAB-54) was included in the combined training set. For the remaining datasets of other disease types, the traditional 10-fold CV were applied, i.e., samples were randomly split into 10 sets, in each round of validation, 9 sets were included in the combined training set and the rest one was included in the combined testing set. Under this strategy, we guarantee the positive training samples are completely independent of the positive testing samples. The negative samples are still somewhat dependent since some of them were generated by traditional 10-fold CV.

Since we have 10 independent datasets, we have 10 rounds of independent validation where each of the 10 datasets was treated as the independent testing set. Within each of the 10 rounds, 10-fold CV was applied in including samples in other disease datasets into the training and testing set. Thus, in total we have 100 rounds of training and validation. The AUC score in Figs 6 and 7 was calculated by the average of the 100 rounds. The error bars in the figures were derived from running the above 100 rounds of validation for 10 times.

### Feature calculation

Some simple gene pre-filtering steps were taken for the original gene expression profiles: 1) genes with mean expression value in lower 25% percentile were removed from each dataset, 2) genes with missing values in more than 20% of the samples in the combined dataset were removed. Cell component profile and residual profile were then calculated for each of the 32 datasets separately, as described in previous sections. All features were Z-transformed before being included into the training and testing set.

It is of note that the cell type-specific expression 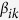 in calculating residual profile and the mean and standard deviation used in Z-transformation has to be derived from control samples of each of 32 datasets. To avoid information leakage, those parameters was derived from control samples within the training set only, and applied to both training and testing set to generate features. For the independent testing dataset (e.g., Obesity_GSE18897) where none of its control samples were included in the training datasets, we used a schema similar to 10-fold CV. In each round of validation, we set aside 9/10 samples to derive the parameters needed in feature generation, and keep the remaining 1/10 samples as the real testing sample.

### Classification

For constructing a classifier of a particular disease type, only samples of that disease type were considered as positive samples, all the rest samples were considered as negative samples including both controls and samples of other diseases. Different classification algorithms were employed. We used R packages *glmnet*, *e1071* and *ranger* for the algorithms of elasticNet, SVM and RF, respectively. We employed an inner loop of 10-fold cross-validation within the training dataset to select the best weighting parameters of alpha and lambda for elasticNet, and to tune the parameter of C for SVM similarly.

### Genes in disease-specific classifiers

We investigated the functional relevance of genes in disease-specific classifiers constructed using residual profiles. In this analysis, we chose DSA as deconvolution method, elasticNet as the classification algorithm and selecting the top-1000 feature as input of elasticNet. To obtain a robust list of genes for a particular disease type, we obtained genes selected by more than half of the corresponding disease-specific classifiers during different runs of cross-validation and bootstrap sampling.

### R package declassification

The gene expression datasets and the algorithms used in this study can be accessible in the R package *decalssification* (http://research.mssm.edu/integrative-network-biology/Software.html). A vignette can also be found on the above website. Briefly, the package includes 32 whole blood gene expression datasets as well as sample labeling and summary descriptions of these datasets. It also includes convenient functions which F can be used to estimate cell components, calculate residual profiles, train the classifiers and etc.

## Additional Information

**How to cite this article**: Wang, L. *et al*. Disease-specific classification using deconvoluted whole blood gene expression. *Sci. Rep.* **6**, 32976; doi: 10.1038/srep32976 (2016).

## Supplementary Material

Supplementary Figures and Tables

Supplementary Table S4

Supplementary Table S5

Supplementary Table S6

Supplementary Table S7

Supplementary Table S8

## Figures and Tables

**Table 1:** Whole blood gene expression profile datasets.

Dataset Name	Case#	Control#	Gene#	Platform	Disease Name&
Aging_GSE33828^28^ $	381	500	23097	Illumina HumanHT-12 V4.0 (GPL10558)	
ASLE_GSE19491^3^	28	17	19982	Illumina HumanHT-12 V3.0 (GPL6947)	ASLE
BacterialPneumonia_GSE20346^29^	26	36	19957	Illumina HumanHT-12 V3.0 (GPL6947)	Pneumonia*
BreastCancer_GSE16443^4^	67	54	16752	ABI Human Genome Survey Microarray V2 (GPL2986)	BreastCancer
ColorectalCancer_E-MEXP-3756^30^	20	20	21049	Affymetrix HG-U133_Plus_2 (GPL570)	ColorectalCancer
CoronaryArteryDiesease_Cathgen_GSE20686^31^	87	52	19749	Agilent-014850 (GPL4133)	CoronaryArteryDiesease
CoronaryArteryDiesease_PREDICT_GSE20686^31^	99	99	19749	Agilent-014850 (GPL4133)	CoronaryArteryDiesease
CRPC_GSE37199^32^	63	31	20618	Affymetrix HG-U133_Plus_2 (GPL570)	CRPC
CRPChighrisk_GSE37199^32^	14	49	20618	Affymetrix HG-U133_Plus_2 (GPL570)	
InfluenzaVaccine_day28_GSE30101^33^	18	18	19982	Illumina HumanHT-12 V3.0 (GPL6947)	
InfluenzaVaccine_day3_GSE30101^33^	18	23	19982	Illumina HumanHT-12 V3.0 (GPL6947)	
InfluenzaVaccine_day7_GSE30101^33^	18	18	19982	Illumina HumanHT-12 V3.0 (GPL6947)	InfluenzaVaccine
InfluenzaVaccine_GSE30101^33^	202	208	19982	Illumina HumanHT-12 V3.0 (GPL6947)	
IntermediateCoronaryArteryDiesease_Cathgen_GSE20686^31^	56	52	19749	Agilent-014850 (GPL4133)	
LTB_test_GSE19491^3^	21	28	19982	Illumina HumanHT-12 V3.0 (GPL6947)	LTB
LTB_training_GSE19491^3^	16	12	19982	Illumina HumanHT-12 V3.0 (GPL6947)	LTB
LungCancer_GSE12771^34^	97	95	24614	Illumina human-6 v2.0 (GPL6102)	LungCancer*
LungCancer_GSE20189^2^	81	80	13211	Affymetrix HG-U133A_2 (GPL571)	LungCancer*
LungCancer_GSE42834^35^	16	118	23871	Illumina HumanHT-12 V4.0 (GPL10558)	LungCancer*
LungCancerStage_GSE20189^2^	29	52	13211	Affymetrix HG-U133A_2 (GPL571)	
MajorDepressiveDisorder_GSE19738^36^	66	66	13331	Agilent-012391 (GPL6848)	MajorDepressiveDisorder
MultipleSclerosis_GSE41850^37^	170	60	17549	Affymetrix Human Exon 1.0 (GPL16209)	MultipleSclerosis
Obesity_GSE18897^38^	20	20	21049	Affymetrix HG-U133_Plus_2 (GPL570)	Obesity*
Obesity_E-MTAB-54^39^	49	25	21049	Affymetrix HG-U133_Plus_2 (GPL570)	Obesity*
Parkinson_GSE6613^40^	50	22	13211	Affymetrix HG-U133A (GPL96)	Parkinson
Pneumonia_GSE42834^35^	24	118	23871	Illumina HumanHT-12 V4.0 (GPL10558)	Pneumonia*
PneumovaxVaccine_day28_GSE30101^33^	15	18	19982	Illumina HumanHT-12 V3.0 (GPL6947)	
PneumovaxVaccine_day3_GSE30101^33^	18	23	19982	Illumina HumanHT-12 V3.0 (GPL6947)	
PneumovaxVaccine_day7_GSE30101^33^	18	18	19982	Illumina HumanHT-12 V3.0 (GPL6947)	PneumovaxVaccine
PneumovaxVaccine_GSE30101^33^	197	208	19982	Illumina HumanHT-12 V3.0 (GPL6947)	
PSLE_GSE19491^3^	82	19	19982	Illumina HumanHT-12 V3.0 (GPL6947)	PSLE
PTB_test_GSE19491^3^	49	28	19982	Illumina HumanHT-12 V3.0 (GPL6947)	TB*
PTB_training_GSE19491^3^	13	12	19982	Illumina HumanHT-12 V3.0 (GPL6947)	TB*
RheumatoidArthritis_GSE17755^41^	112	45	14358	Hitachisoft AceGene Human Oligo Chip (GPL1291)	RheumatoidArthritis
Sarcoid_GSE42834^35^	83	118	23871	Illumina HumanHT-12 V4.0 (GPL10558)	Sarcoid
Schizophrenia_GSE38485^42^	106	96	19969	Illumina HumanHT-12 V3.0 (GPL6947)	Schizophrenia
SevereInfluenza_GSE20346^29^	19	36	19957	Illumina HumanHT-12 V3.0 (GPL6947)	SevereInfluenza
SleepRestriction_16.5_GSE39445^43^	22	23	19541	Agilent-026817 (GPL15331)	
SleepRestriction_25.5_GSE39445^43^	23	22	19541	Agilent-026817 (GPL15331)	
SleepRestriction_34.5_GSE39445^43^	20	20	19541	Agilent-026817 (GPL15331)	
SleepRestriction_7.5_GSE39445^43^	23	22	19541	Agilent-026817 (GPL15331)	SleepRestriction
SleepRestriction_GSE39445^43^	212	215	19541	Agilent-026817 (GPL15331)	
STAPH_GSE19491^3^	40	23	19982	Illumina HumanHT-12 V3.0 (GPL6947)	STAPH
STILL_GSE19491^3^	31	22	19982	Illumina HumanHT-12 V3.0 (GPL6947)	STILL
STREP_GSE19491^3^	12	23	19982	Illumina HumanHT-12 V3.0 (GPL6947)	STREP
TB_GSE42834^35^	40	118	23871	Illumina HumanHT-12 V4.0 (GPL10558)	TB*

^&^Datasets with empty disease names were not included in building disease-specific classifiers (see Methods for details).

^*^10 independent datasets used in evaluating performance of disease-specific classifiers.

^$^For the dataset of Aging_GSE33828, samples were split into case (old) and control(young) group at the cutoff age of 60 years old (this cutoff is empirically chosen while to make the case and control group of similar size).
