# Supplementary material for: Disease-specific classification using deconvoluted whole blood gene expression
Source: Sci Rep. 2016 Sep 6;6:32976. doi: 10.1038/srep32976 (PMC5011717; doi:10.1038/srep32976)
Supplement: Supplementary Figures and Tables [file srep32976-s1.pdf]

Supplementary Materials for

Disease-specific classification using deconvoluted whole blood gene expression

Li Wang[1,2], William K. Oh[3], Jun Zhu[1,2,3,*]

[1]Icahn Institute for Genomics and Multiscale Biology; [2]Department of Genetics and Genomic Sciences; [3]The Tisch Cancer Institute, Division of Hematology and Medical Oncology; Icahn School of Medicine at Mount Sinai, NY, USA.

Supplementary Tables

Table S1 Availability of age, gender and race information for individual sample in each dataset.

	age	gender	race
Aging_GSE33828	NO	YES	NO
ASLE_GSE19491	YES	YES	YES
BacterialPneumonia_GSE20346	NO	NO	NO
BreastCancer_GSE16443	YES	NO	NO
CRPC_GSE37199	NO	NO	NO
CRPChighrisk_GSE37199	NO	NO	NO
ColorectalCancer_E-MEXP-3756	NO	NO	NO
CoronaryArteryDiesease_Cathgen_GSE20686	NO	NO	NO
CoronaryArteryDiesease_PREDICT_GSE20686	YES	YES	NO
InfluenzaVaccine_GSE30101	YES	YES	YES
InfluenzaVaccine_day28_GSE30101	YES	YES	YES
InfluenzaVaccine_day3_GSE30101	YES	YES	YES
InfluenzaVaccine_day7_GSE30101	YES	YES	YES
IntermediateCoronaryArteryDiesease_Cathgen_GSE20686	NO	NO	NO
LTB_test_GSE19491	YES	YES	YES
LTB_training_GSE19491	YES	YES	YES
LungCancerStage_GSE20189	NO	NO	NO
LungCancer_GSE12771	NO	NO	NO
LungCancer_GSE20189	NO	NO	NO
LungCancer_GSE42834	NO	YES	YES
MajorDepressiveDisorder_GSE19738	YES	YES	NO
MultipleSclerosis_GSE41850	NO	YES	NO
Obesity_GSE18897	NO	YES	NO
Obesity_E-MTAB-54	YES	YES	NO
PSLE_GSE19491	YES	YES	YES
PTB_test_GSE19491	YES	YES	YES
PTB_training_GSE19491	YES	YES	YES
Parkinson_GSE6613	NO	NO	NO
Pneumonia_GSE42834	NO	YES	YES
PneumovaxVaccine_GSE30101	YES	YES	YES
PneumovaxVaccine_day28_GSE30101	YES	YES	YES
PneumovaxVaccine_day3_GSE30101	YES	YES	YES
PneumovaxVaccine_day7_GSE30101	YES	YES	YES
RheumatoidArthritis_GSE17755	YES	YES	NO
STAPH_GSE19491	YES	YES	YES
STILL_GSE19491	YES	YES	YES

STREP_GSE19491	YES	YES	YES
Sarcoid_GSE42834	NO	YES	YES
Schizophrenia_GSE38485	YES	YES	NO
SevereInfluenza_GSE20346	NO	NO	NO
SleepRestriction_16.5_GSE39445	NO	NO	NO
SleepRestriction_25.5_GSE39445	NO	NO	NO
SleepRestriction_34.5_GSE39445	NO	NO	NO
SleepRestriction_7.5_GSE39445	NO	NO	NO
SleepRestriction_GSE39445	NO	NO	NO
TB_GSE42834	NO	YES	YES

Table S2 Demographic characteristic for case samples in each dataset

	Age (median)	Female (Fraction)	White (Fraction)
Aging_GSE33828	64.2	0.55	NA
ASLE_GSE19491	36	0.86	0.54
BacterialPneumonia_GSE20346	63	0.5	NA
BreastCancer_GSE16443	57	1	NA
ColorectalCancer_E-MEXP-3756	56.6	0.55	NA
CoronaryArteryDiesease_Cathgen_GSE20686	63	0.33	0.69
CoronaryArteryDiesease_PREDICT_GSE20686	64	0.24	0.93
CRPC_GSE37199	NA	NA	NA
CRPChighrisk_GSE37199	NA	NA	NA
InfluenzaVaccine_day28_GSE30101	30.5	0.5	0.61
InfluenzaVaccine_day3_GSE30101	30.5	0.5	0.5
InfluenzaVaccine_day7_GSE30101	30.5	0.5	0.5
InfluenzaVaccine_GSE30101	32	0.50	0.52
IntermediateCoronaryArteryDiesease_Cathgen_GSE20686	63	0.33	0.69
LTB_test_GSE19491	33	0.52	0.14
LTB_training_GSE19491	31	0.5	0.13
LungCancer_GSE12771	63	0.37	NA
LungCancer_GSE20189	67	0.49	NA
LungCancer_GSE42834	59	0.38	0.94
LungCancerStage_GSE20189	67	0.49	NA
MajorDepressiveDisorder_GSE19734	43	0.64	NA
MultipleSclerosis_GSE41850	44	0.69	NA
Obese_GSE18897	52.2	0.6	NA
Obesity_E-MTAB-54	48	0.51	NA
Parkinson_GSE6613	69.4	0.22	NA
Pneumonia_GSE42834	63	0.37	0.46

PneumovaxVaccine_day28_GSE30101	31	0.53	0.87
PneumovaxVaccine_day3_GSE30101	29	0.5	0.78
PneumovaxVaccine_day7_GSE30101	29	0.5	0.78
PneumovaxVaccine_GSE30101	29	0.50	0.80
PSLE_GSE19491	16	0.84	0.15
PTB_test_GSE19491	33	0.33	0.33
PTB_training_GSE19491	29	0.46	0.23
RheumatoidArthritis_GSE17755	54	0.83	NA
Sarcoid_GSE42834	47	0.53	0.40
Schizophrenia_GSE38485	40.5	0.28	NA
SevereInfluenza_GSE20346	33	0.75	NA
SleepRestriction_16.5_GSE39445	27.5	0.46	NA
SleepRestriction_25.5_GSE39445	27.5	0.46	NA
SleepRestriction_34.5_GSE39445	27.5	0.46	NA
SleepRestriction_7.5_GSE39445	27.5	0.46	NA
SleepRestriction_GSE39445	27.5	0.46	NA
STAPH_GSE19491	8	0.5	0.23
STILL_GSE19491	38	0.48	0.74
STREP_GSE19491	5	0.5	0.33
TB_GSE42834	39	0.46	0.28

Table S3 Number of datasets inside and outside of the tight cluster in Figure 1 in each disease cateogory. We excluded some longitudinal datasets (those with the column of disease name blank in Table 1 except for Aging_GSE33828) in order to avoid multiple count of the same disease dataset.

	outside	inside
Aging	0	1
Cardiovascular disease	1	1
Cancer	1	5
Inflammatory and infectious diseases	4	11
Neuronal disease	4	0
Non-morbid condition (vaccine/sleep deprivation)	3	0
Metabolic disease	2	0

Supplementary Figures

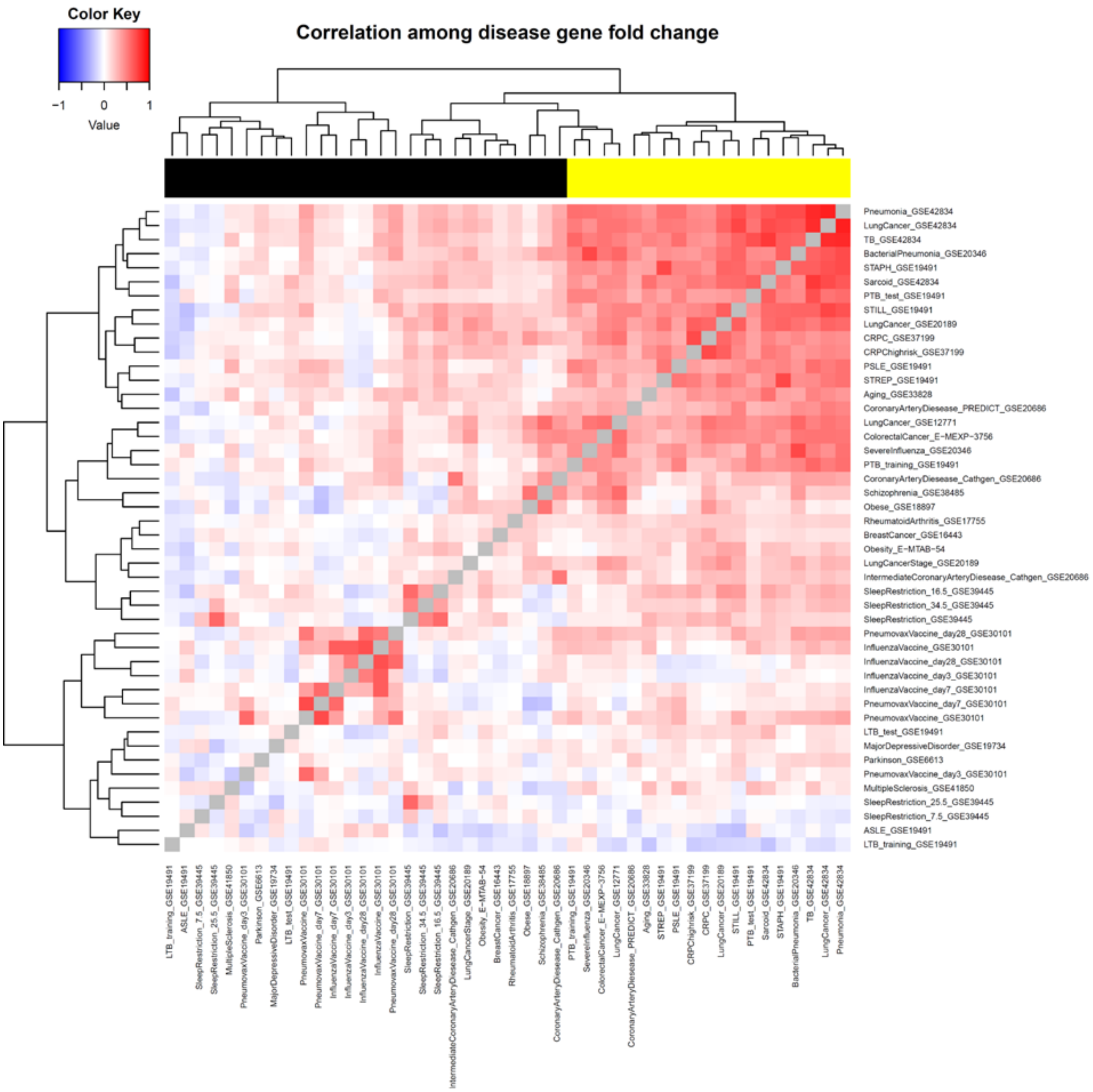

Figure S1 Correlation among gene expression changes of diverse disease signature genes after correcting for the effect of age, gender and race. Yellow color in the side bar indicates the dataset is inside the tight cluster in Figure 1

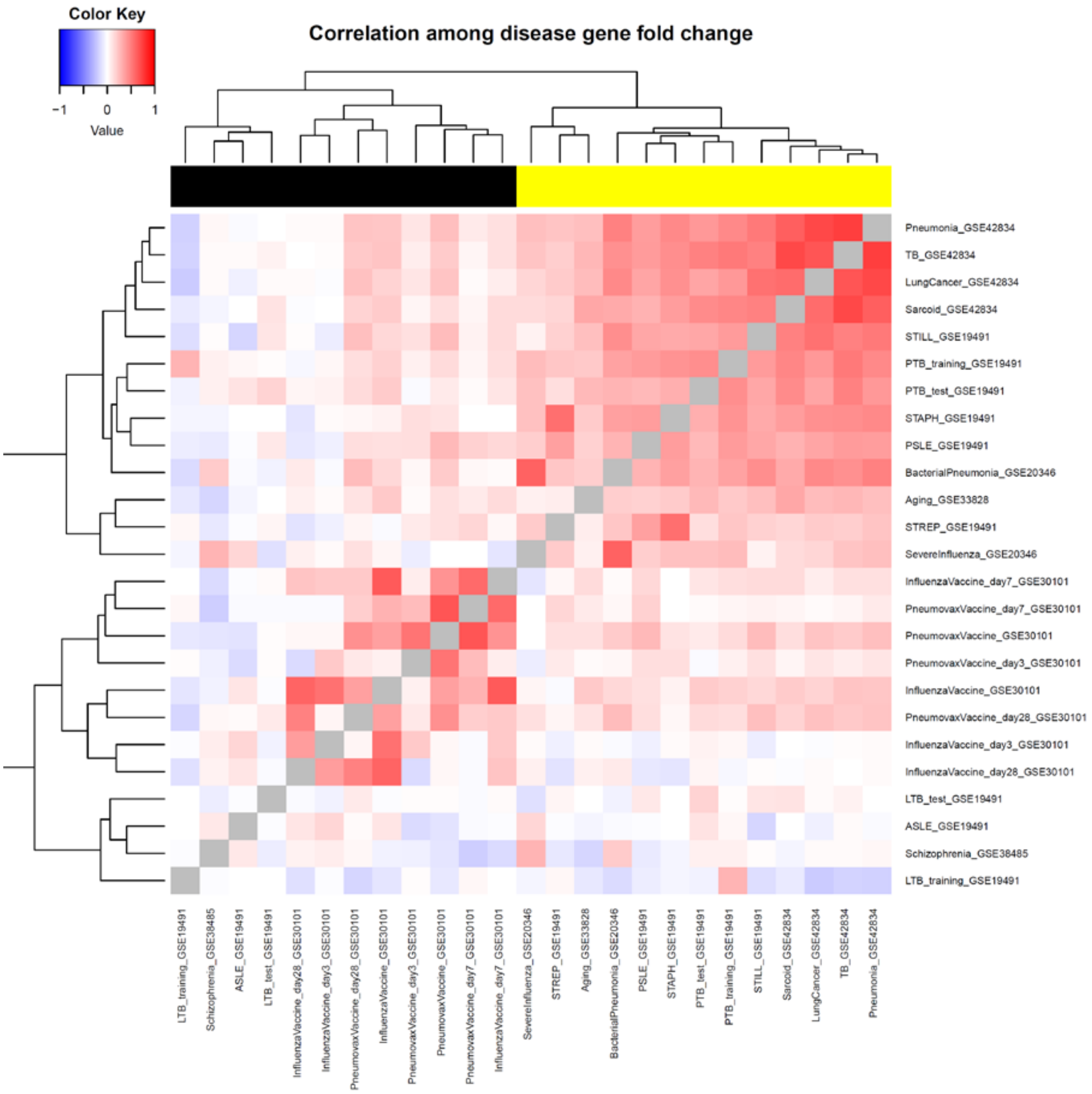

Figure S2 Correlation among gene expression changes for 25 datasets generated on illumina humanHT-12. Yellow color in the side bar indicates the dataset is inside the tight cluster in Figure 1.

A)

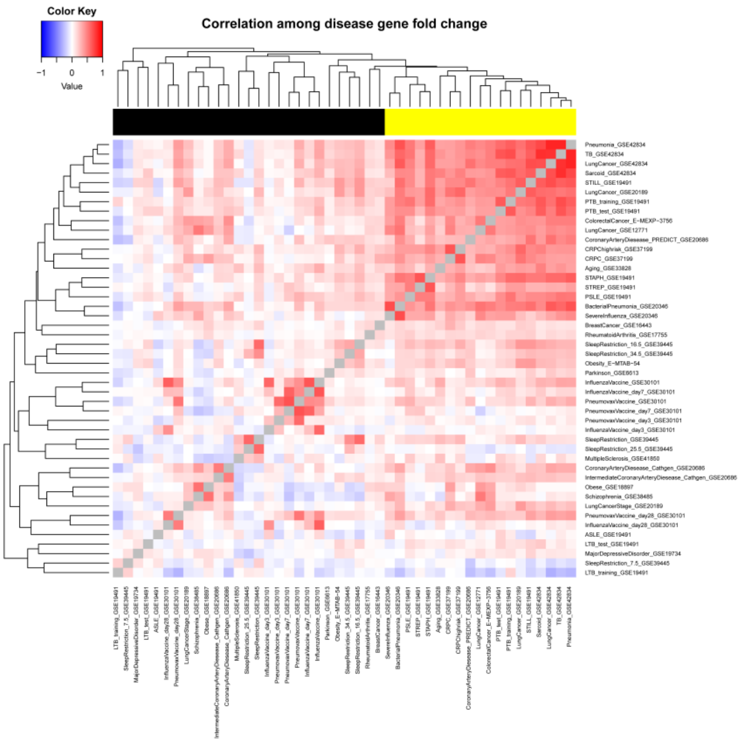

B)

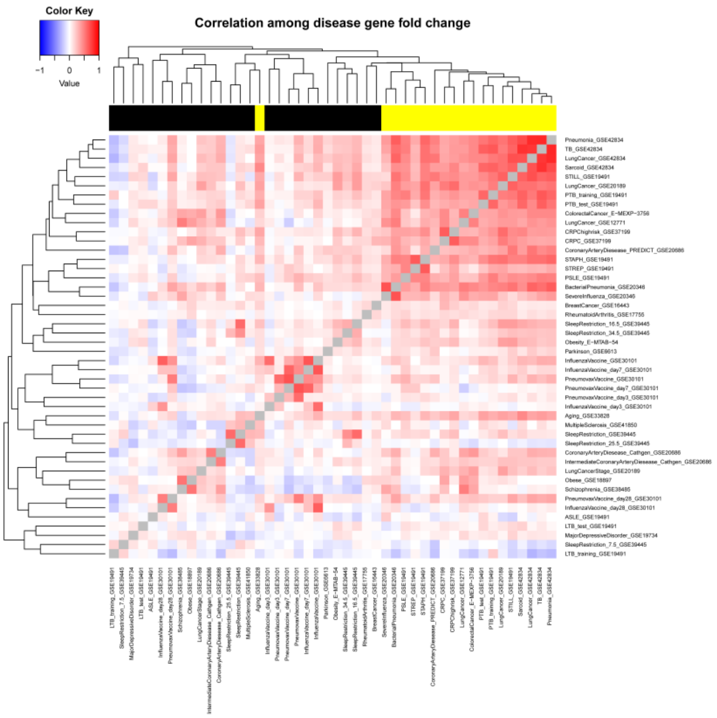

Figure S3 Correlation among expression change profiles of disease informative genes which were pooled from top 300 (A) and 500 (B) differentially expressed genes in each dataset. Yellow color in the side bar indicates the dataset is inside the tight cluster in Figure 1

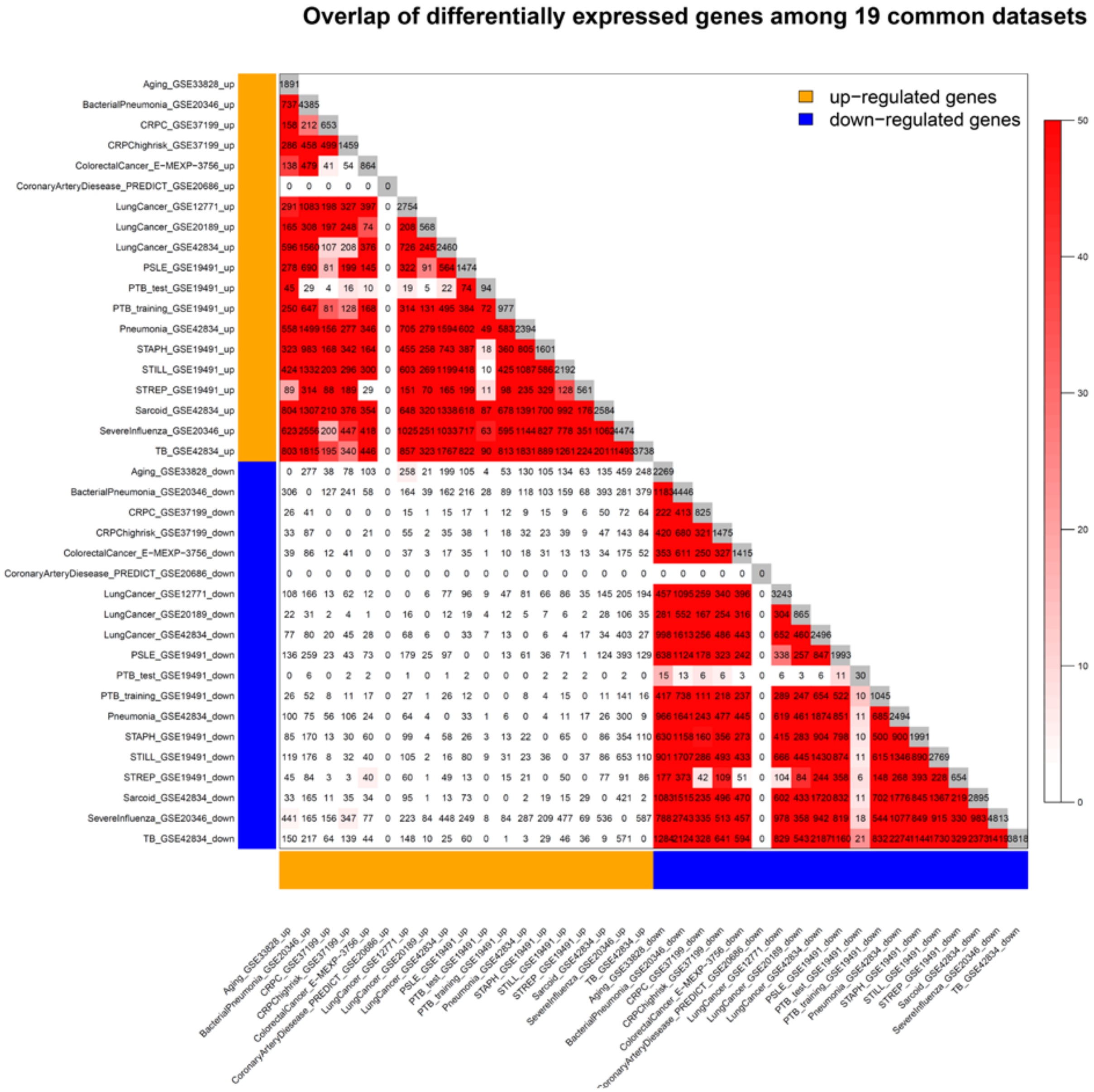

Figure S4 Overlaps of differentially expressed genes among 19 common datasets. Each row or column corresponds to one differentially expressed gene list. Orange color in the side bar represents significantly up-regulated gene list (FDR<0.1 using R package limma), and blue color represents significantly down-regulated gene list. Numbers in the table indicate gene counts in the intersection of two gene lists. Coloring of the table encodes –log(p), with p being the Fisher's exact test p-value for the overlap of the two modules.

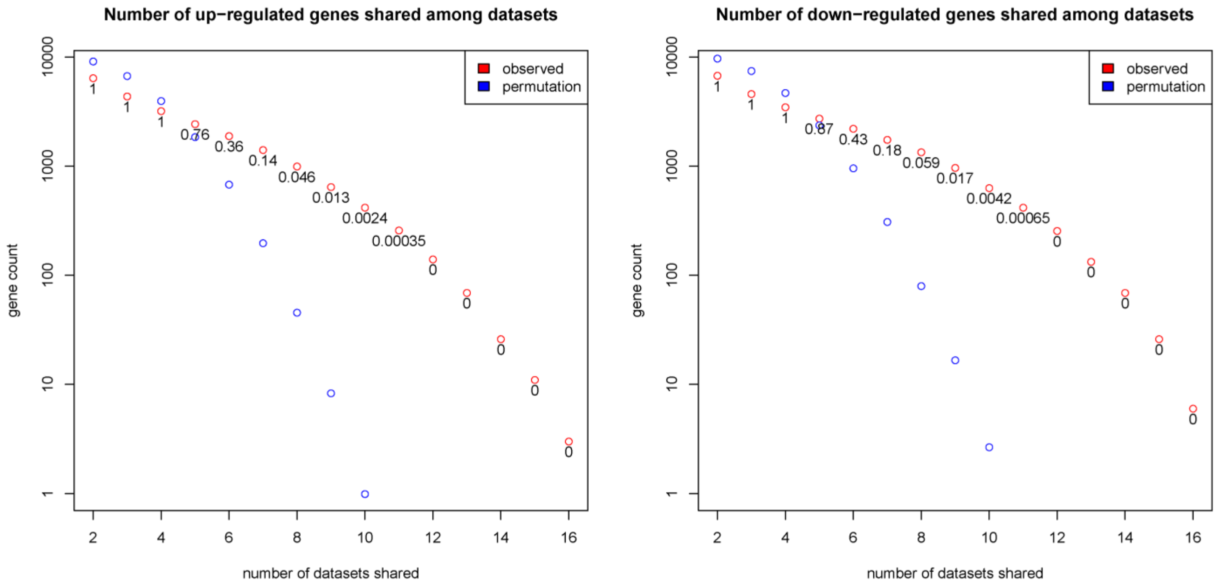

Figure S5 Numbers of significantly differentially expressed genes shared among the 19 similar datasets. Y axes represents the number of genes shared by >= n datasets (n as indicates by X axes). Red color indicates the observed gene counts, and blue color indicates the gene counts under permutation. The number under the red dots represents the FDR=permutation/observed.

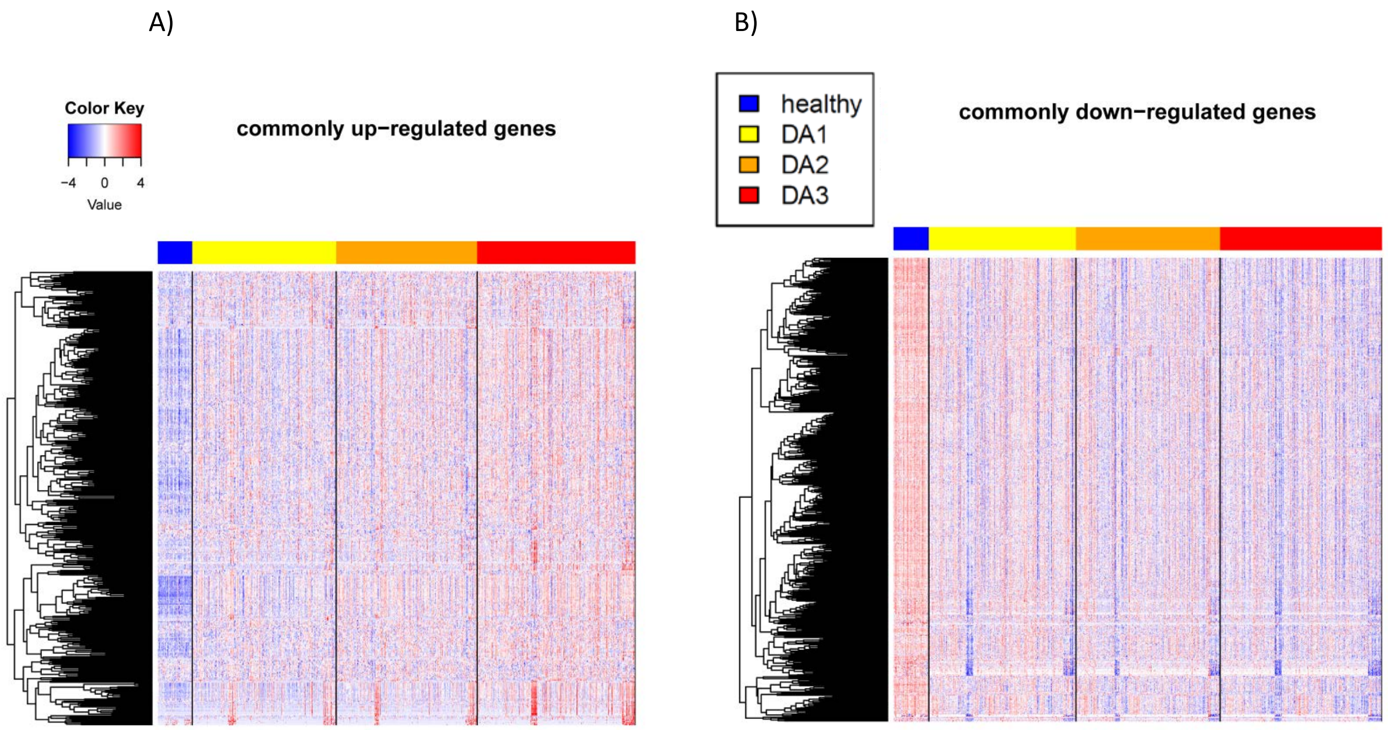

Figure S6 Heatmap of the commonly up-regulated (A) and down-regulated (B) genes in SLE_GSE65391. Color bars indicate the disease activity of patient samples or if it is a control. DA1, DA2 and DA3 represents three disease activity categories with DA3 the most severe and DA1 the mildest.

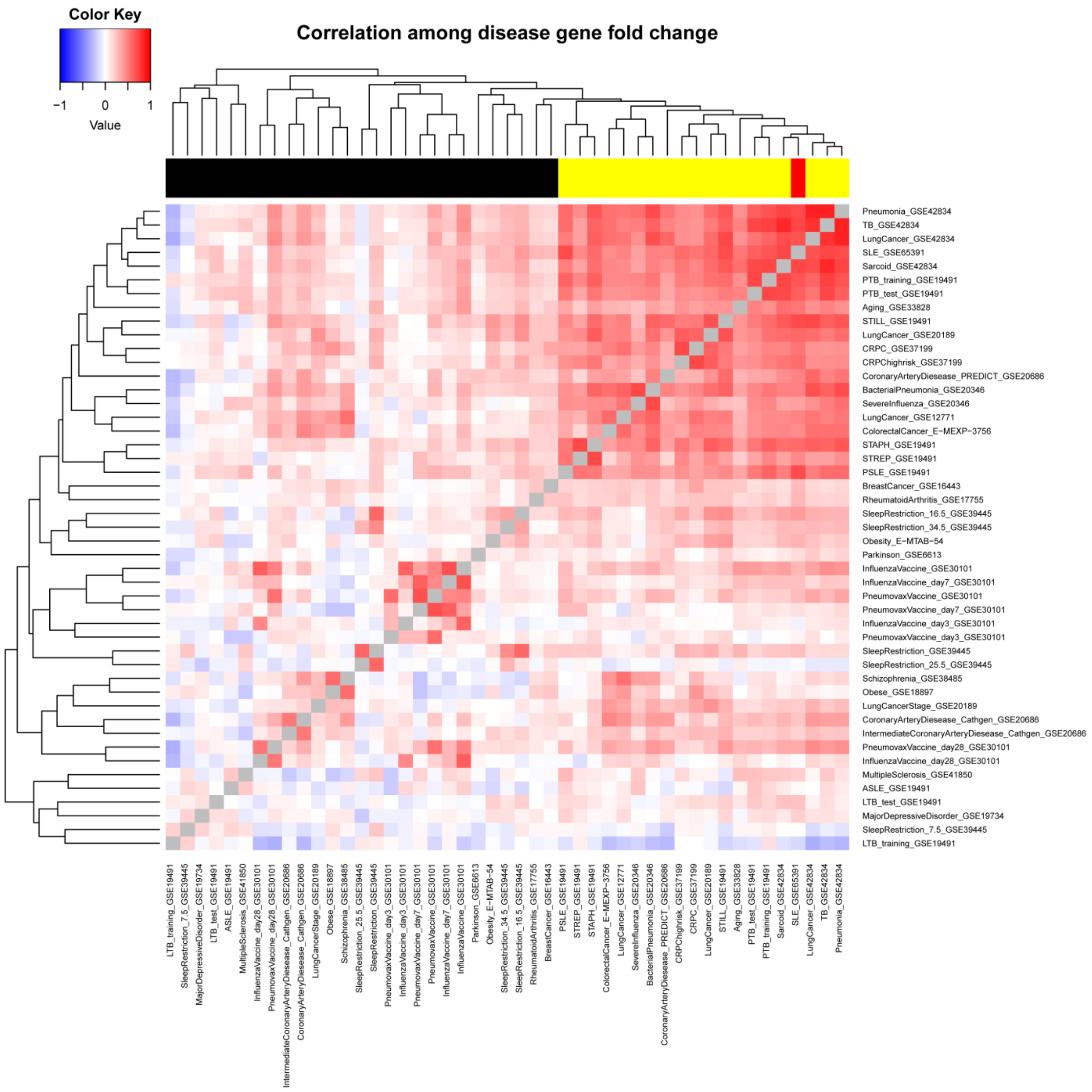

Figure S7 Correlation among gene expression changes of diverse disease signature when the prospective dataset SLE_GSE65391 were included. Yellow color in the side bar represents the dataset inside the tight cluster in Figure 1, and the red color represents the prospective dataset SLE_GSE65391.

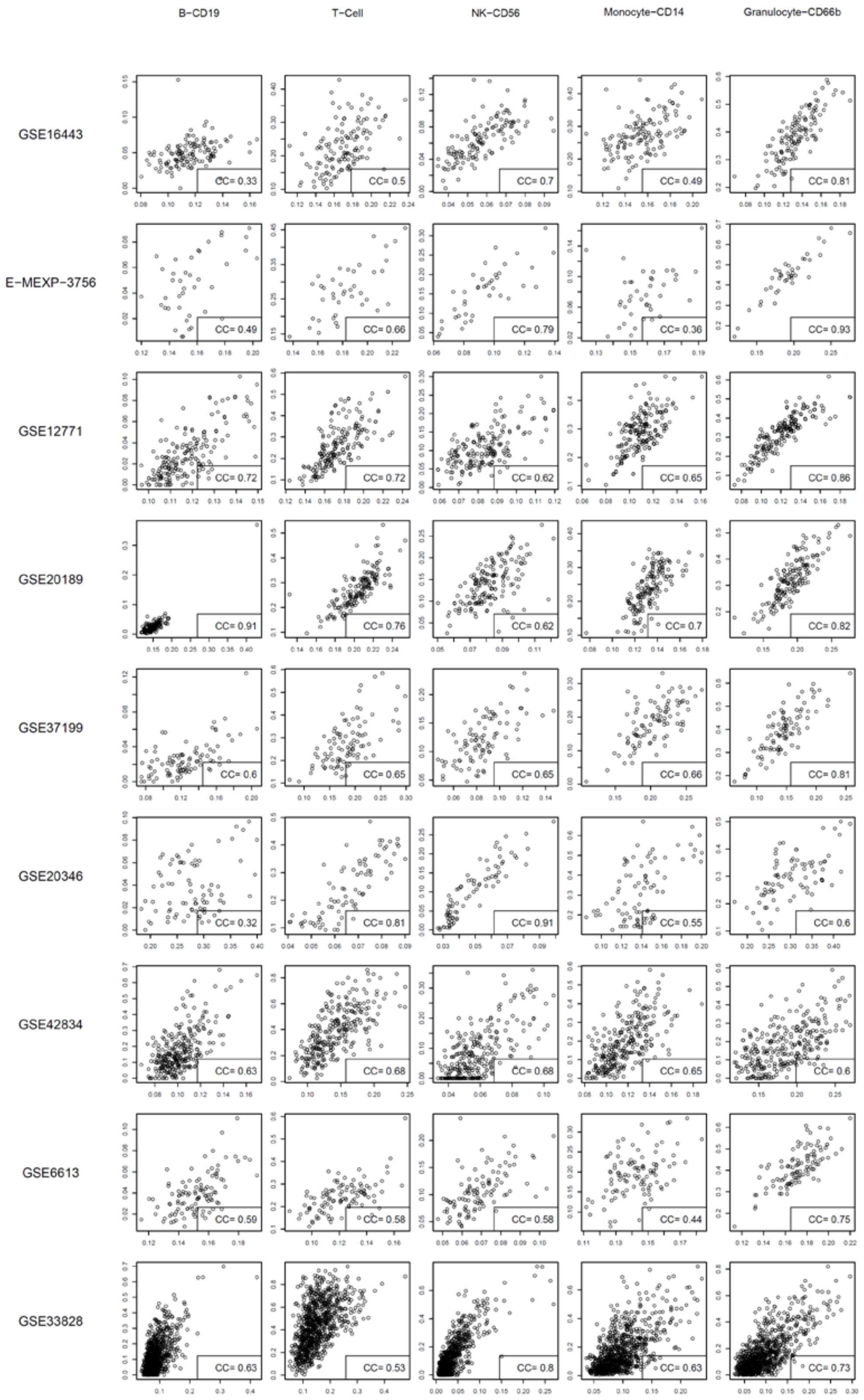
B−CD19
T−Cell
NK−CD56
Monocyte−CD14
Granulocyte−CD66b
GSE16443
CC= 0.33
CC= 0.5
CC= 0.7
CC= 0.49
CC= 0.81
E−MEXP−3756
CC= 0.49
CC= 0.66
CC= 0.79
CC= 0.36
CC= 0.93
GSE12771
CC= 0.72
CC= 0.72
CC= 0.62
CC= 0.65
CC= 0.86
GSE20189
CC= 0.91
CC= 0.76
CC= 0.62
CC= 0.7
CC= 0.82
GSE37199
CC= 0.6
CC= 0.65
CC= 0.65
CC= 0.66
CC= 0.81
GSE20346
CC= 0.32
CC= 0.81
CC= 0.91
CC= 0.55
CC= 0.6
GSE42834
CC= 0.63
CC= 0.68
CC= 0.68
CC= 0.65
CC= 0.6
GSE6613
CC= 0.59
CC= 0.58
CC= 0.58
CC= 0.44
CC= 0.75
GSE33828
CC= 0.63
CC= 0.53
CC= 0.8
CC= 0.63
CC= 0.73

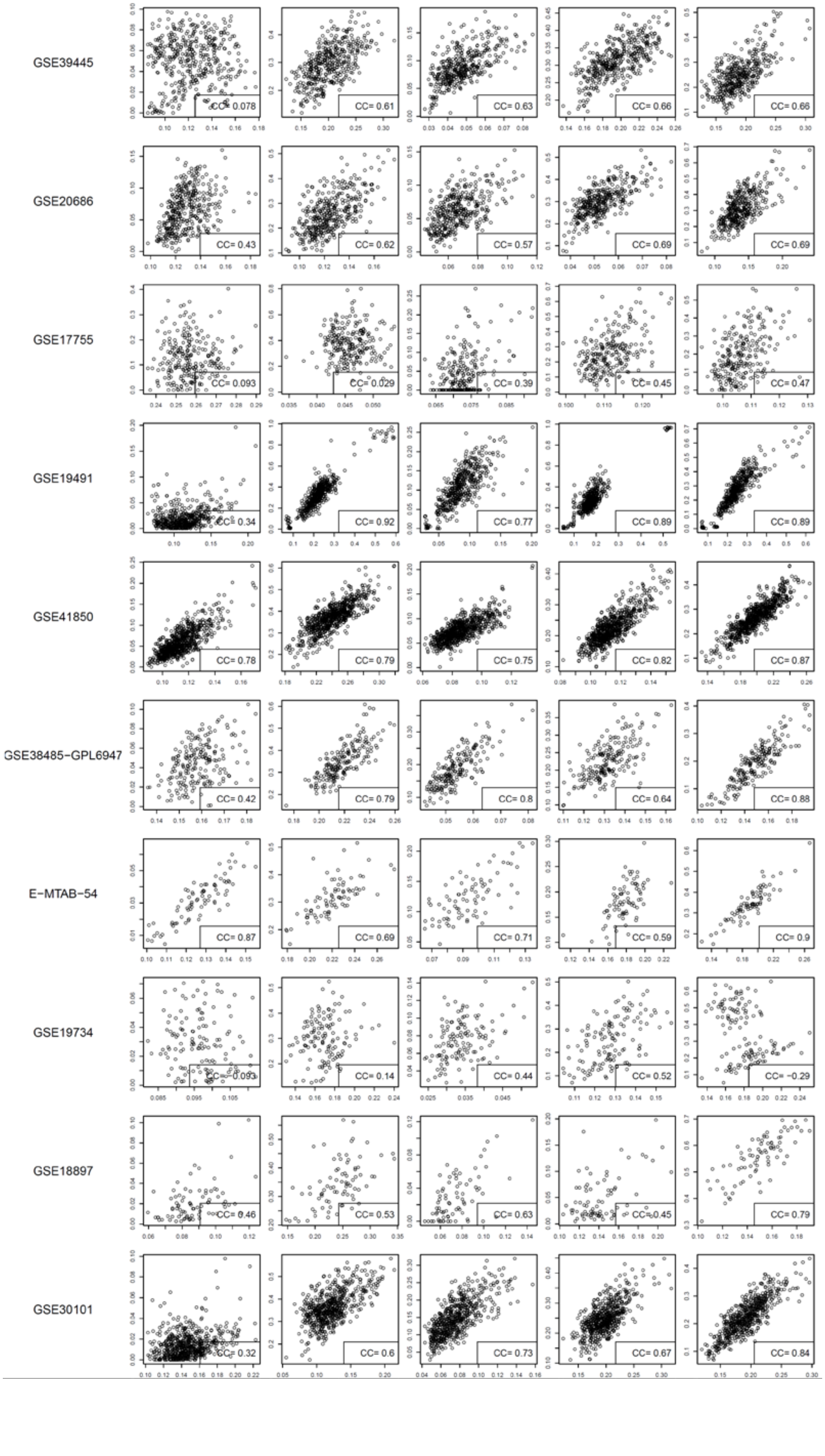
GSE39445
CC= 0.078
CC= 0.61
CC= 0.63
CC= 0.66
CC= 0.66
GSE20686
CC= 0.43
CC= 0.62
CC= 0.57
CC= 0.69
CC= 0.69
GSE17755
CC= 0.093
CC= 0.029
CC= 0.39
CC= 0.45
CC= 0.47
GSE19491
CC= 0.34
CC= 0.92
CC= 0.77
CC= 0.89
CC= 0.89
GSE41850
CC= 0.78
CC= 0.79
CC= 0.75
CC= 0.82
CC= 0.87
GSE38485-GPL6947
CC= 0.42
CC= 0.79
CC= 0.8
CC= 0.64
CC= 0.88
E-MTAB-54
CC= 0.87
CC= 0.69
CC= 0.71
CC= 0.59
CC= 0.9
GSE19734
CC= 0.093
CC= 0.14
CC= 0.44
CC= 0.52
CC= -0.29
GSE18897
CC= 0.46
CC= 0.53
CC= 0.63
CC= 0.45
CC= 0.79
GSE30101
CC= 0.32
CC= 0.6
CC= 0.73
CC= 0.67
CC= 0.84

Figure S8 Plots of cell frequency estimation by DSA (X-axis) and CIBERSORT (Y-axis). Each dot represents one sample. Each row represents one dataset, and each column represents one cell component. Since CIBERSORT outputs detailed frequency of cells at different status while DSA doesn't, we combined the corresponding estimation from CIBERSORT, e.g., the frequency of B-cell naïve, B-cell memory and plasma cells were combined to compare with that of B-CD19 in DSA.

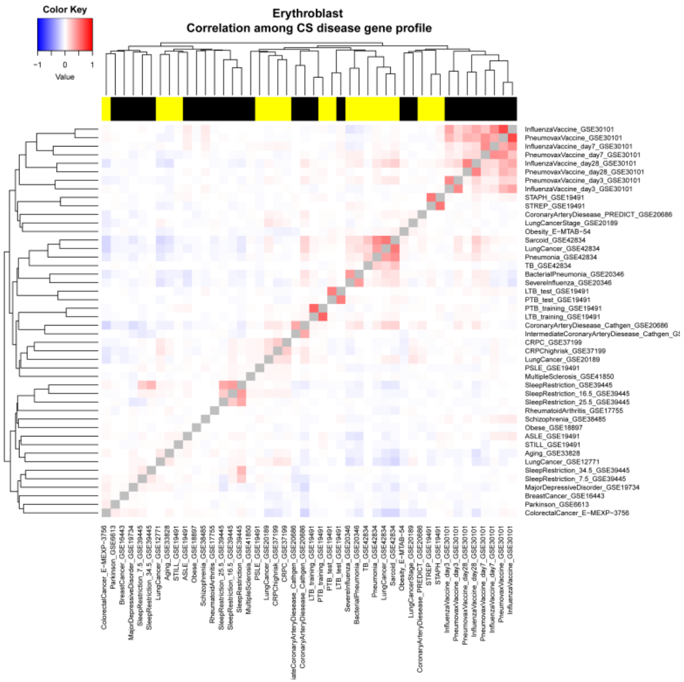

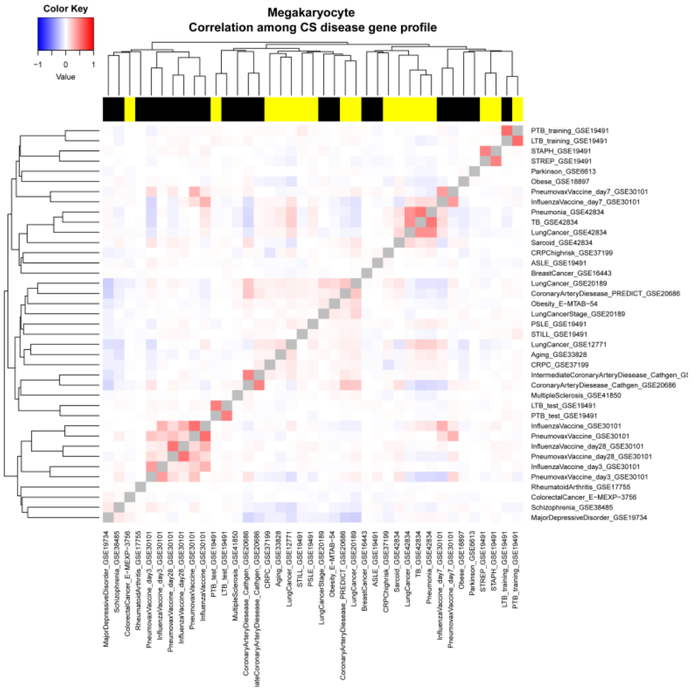

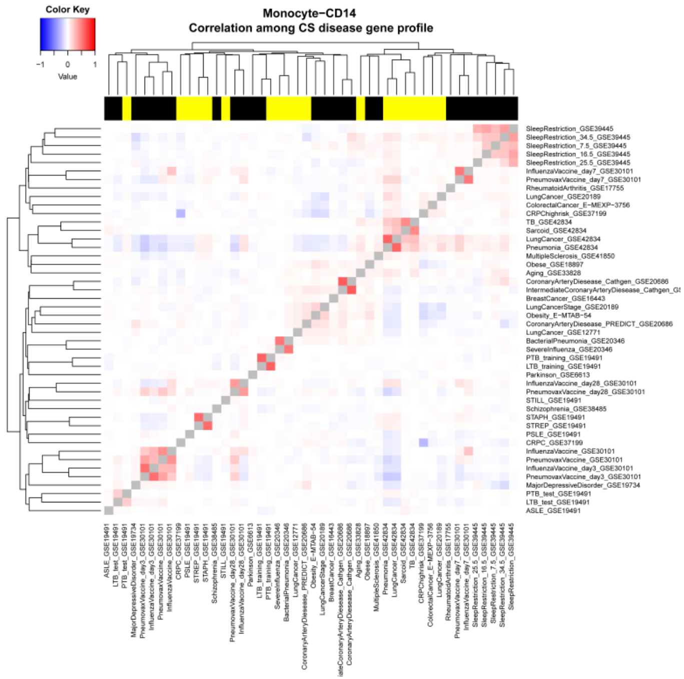

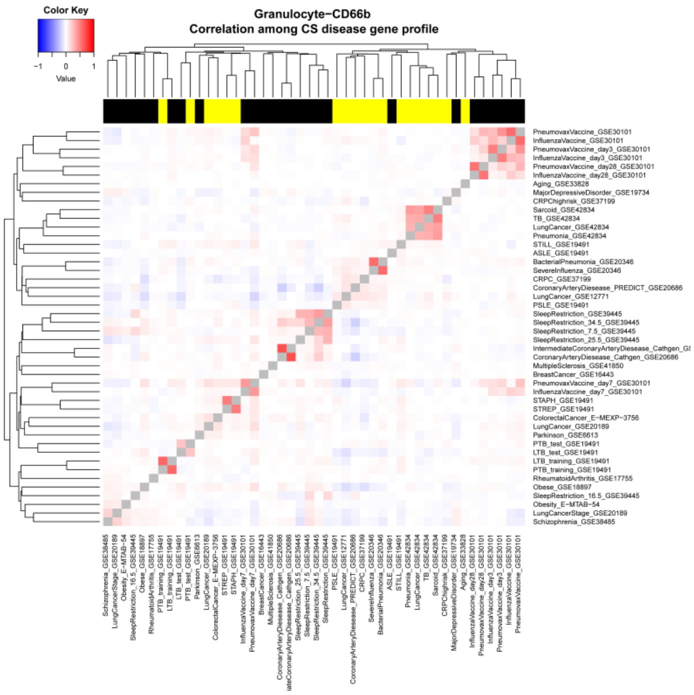

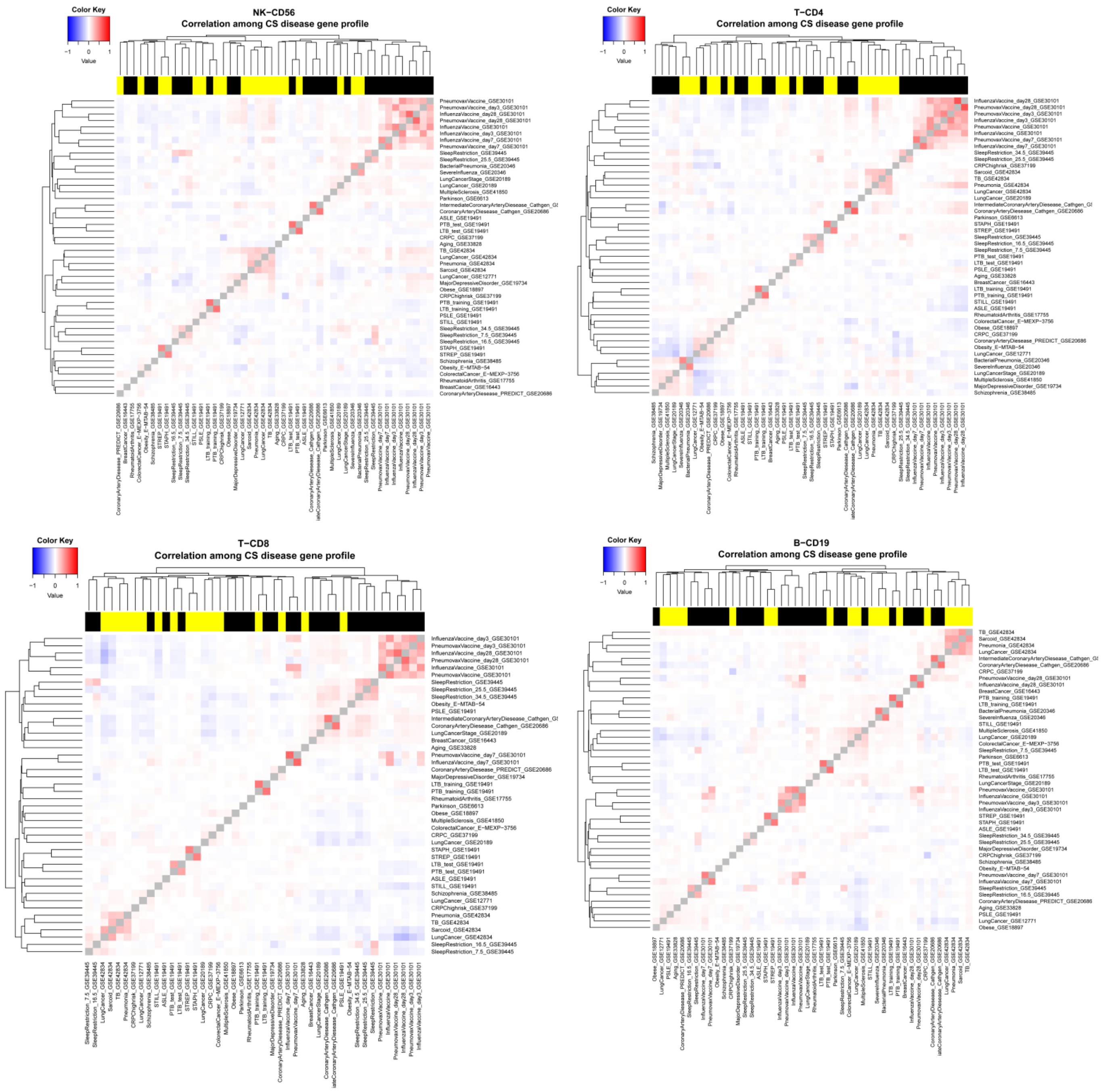

Figure S9 Disease similarity based on cell type-specific expression difference. For each disease dataset and each cell type, csSAM algorithm outputs a t-statistic profile representing how genes are differentially expressed in this cell type and in this disease dataset. Each heatmap above represents one cell type, and each cell in the heatmap represents correlation coefficient of the t-statistic profile of the two corresponding disease datasets. (Please refer to the method section of similarity among disease gene signatures in the main manuscript about details in calculating correlation coefficients). Yellow color in the side bar indicates the dataset is inside the tight cluster in Figure 1.

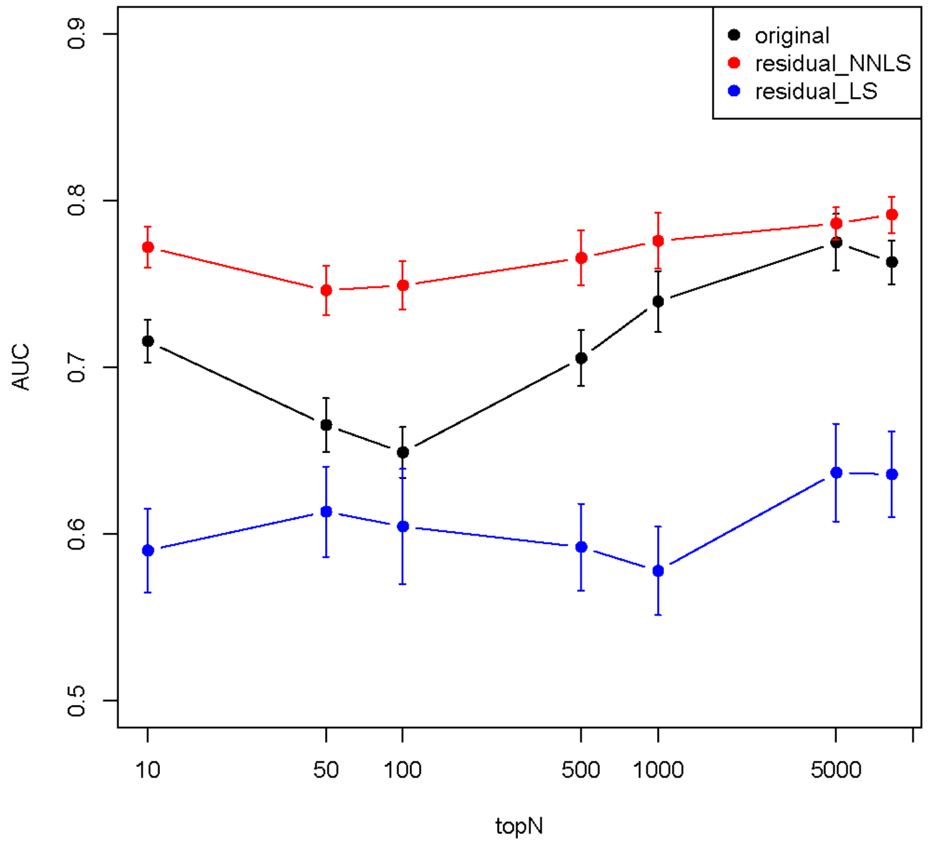

Figure S10 Performance of disease-specific classifiers based on the original whole blood gene expression profile or the residual expression profile. The cell type specific expression profiles used in computing residual expression profile was calculated in two different ways, i.e., non-negative least square (NNLS) or ordinal least square (LS). Please refer to the legend of Figure 6 for more details.
